# Multi-omics reveals immune features in immune and non-immune cells, an IFN-γ/IFN-α-B2M positive feedback loop, and targeted metabolic therapy in multiple myeloma

**DOI:** 10.3389/fimmu.2025.1575079

**Published:** 2025-09-08

**Authors:** Chen Li, Yaping Liao, Lingyun Xu, Yan Chen

**Affiliations:** ^1^ Department of Hematology, The Eighth Affiliated Hospital, Sun Yat-sen University, Shenzhen, China; ^2^ Department of Hematology, Fuyang People’s Hospital (The Affiliated Fuyang People’s Hospital of Anhui Medical University), Fuyang, China

**Keywords:** multiple myeloma, multi-omics atlas, non-immune cells, signaling pathway, metabolic reprogramming-targeted

## Abstract

Multiple myeloma (MM) is highly heterogeneous, with relapse occurring in the majority of cases, and recent advancements in single-cell RNA sequencing (scRNA-seq), sc-metabolism profiling, and bulk RNA-seq have facilitated the identification of cell subpopulations and metabolic reprogramming at the single-cell level, uncovering novel molecular mechanisms. This study aims to establish a multi-omics atlas of MM, characterizing the cell subpopulations and signaling pathways that drive immune evasion and disease progression. Additionally, sc-metabolic profiling identifies reprogramming patterns and informs therapeutic screening. We integrated scRNA-seq and bulk RNA-seq data using R to analyze immune and non-immune cell features and pathways in MM. Metabolic reprogramming was assessed via sc-metabolic profiling, and drug candidates were screened through multi-omics integration, with efficacy evaluated *in vitro* using CCK-8 assays, flow cytometry, Western blotting, and CalcuSyn software. Novel MM subpopulations were identified, including myeloma-activated hematopoietic stem cells and ISG15+ B cells, which correlated with survival and were validated by multiplex immunofluorescence. IFN-γ is primarily secreted by effector memory CD8+T cells, and IFN-α is primarily secreted by non-classical monocytes, driving an IFN-γ/α-B2M feedback loop. Multi-omics identified four drug candidates, each demonstrating anti-tumor effects against myeloma cell lines.

## 1 Introduction

Multiple myeloma (MM) remains an incurable malignancy with a high risk of recurrence, despite advancements in therapeutic strategies (1, 2). Current treatments, including proteasome inhibitors, immunomodulatory regimens, and haematopoietic cell transplants, have led to significant improvements in patient outcomes. However, challenges such as relapse, drug resistance, and treatment-associated toxicity continue to limit long-term efficacy (1–3). Consequently, a deeper understanding of the tumor microenvironment (TME) and the underlying pathogenesis of MM is essential for the discovery of novel and more effective treatment approaches.

Recent breakthroughs in high-throughput technologies, such as single-cell RNA sequencing (scRNA-seq), bulk RNA sequencing (RNA-seq), and single-cell metabolic profiling, have revolutionized our ability to investigate MM at unprecedented resolution. ScRNA-seq enables detailed transcriptomic analysis at the single-cell level, allowing for the identification of heterogeneous cell populations within the TME. Bulk RNA-seq, in contrast, provides robust survival data and gene expression profiles, while single-cell metabolism profiling offers critical insights into the metabolic alterations that underpin tumor progression. The integration of these powerful technologies facilitates the creation of a comprehensive multi-omics atlas, which provides a more nuanced understanding of MM at single-cell resolution.

Despite the wealth of knowledge generated by previous scRNA-seq studies on MM, these investigations have primarily focused on specific disease stages or isolated cell populations. MM, however, is a highly heterogeneous disease characterized by clonal evolution, which necessitates a broader approach to fully understand its complexities. To address this, the current study integrates four datasets comprising 113 participants, including healthy controls and individuals at various stages of disease progression. This analysis represents the largest single-cell multi-omics study of MM to date.

Through this multi-omics approach, we identified and validated novel subpopulations within the MM TME. These findings were corroborated by multiplex immunofluorescence, offering new insights into the TME’s role in disease progression. Furthermore, this study provides potential therapeutic targets, advancing our understanding of MM and opening the door for future therapeutic strategies.

## 2 Materials and methods

Complete details are in **Supplementary File 1**. scRNA-seq datasets were integrated, quality-controlled, and analyzed for clustering, annotation, cell-cell communication, and pseudo-time dynamics. Functional enrichment and transcription factor networks identified key biological pathways. Metabolic activity was quantified using scMetabolism R and visualized with heatmaps/nomograms. GSEA and GSVA revealed pathways related to plasma cell malignancy, and key enzyme expression in glucose, lipid, and glutamine metabolism was assessed. Bulk RNA-seq analysis used CIBERSORTx for cell abundance quantification, with KM survival analysis. mIF on tissue sections validated cell subpopulations. Drug screening identified galloflavin, CB-839, NAC, and EGCG inhibitors, with effects on KM3 cell proliferation assessed through CCK-8 assays, flow cytometry, and Western blotting. The validation cohort consisted of MM patients and healthy controls, with data on β-2M, Ferritin, serum Fe, TF, TSAT, IFN-γ, IL-6, and CRP.

## 3 Results

### 3.1 Integrated multi-omics analysis

**Figure 1:**
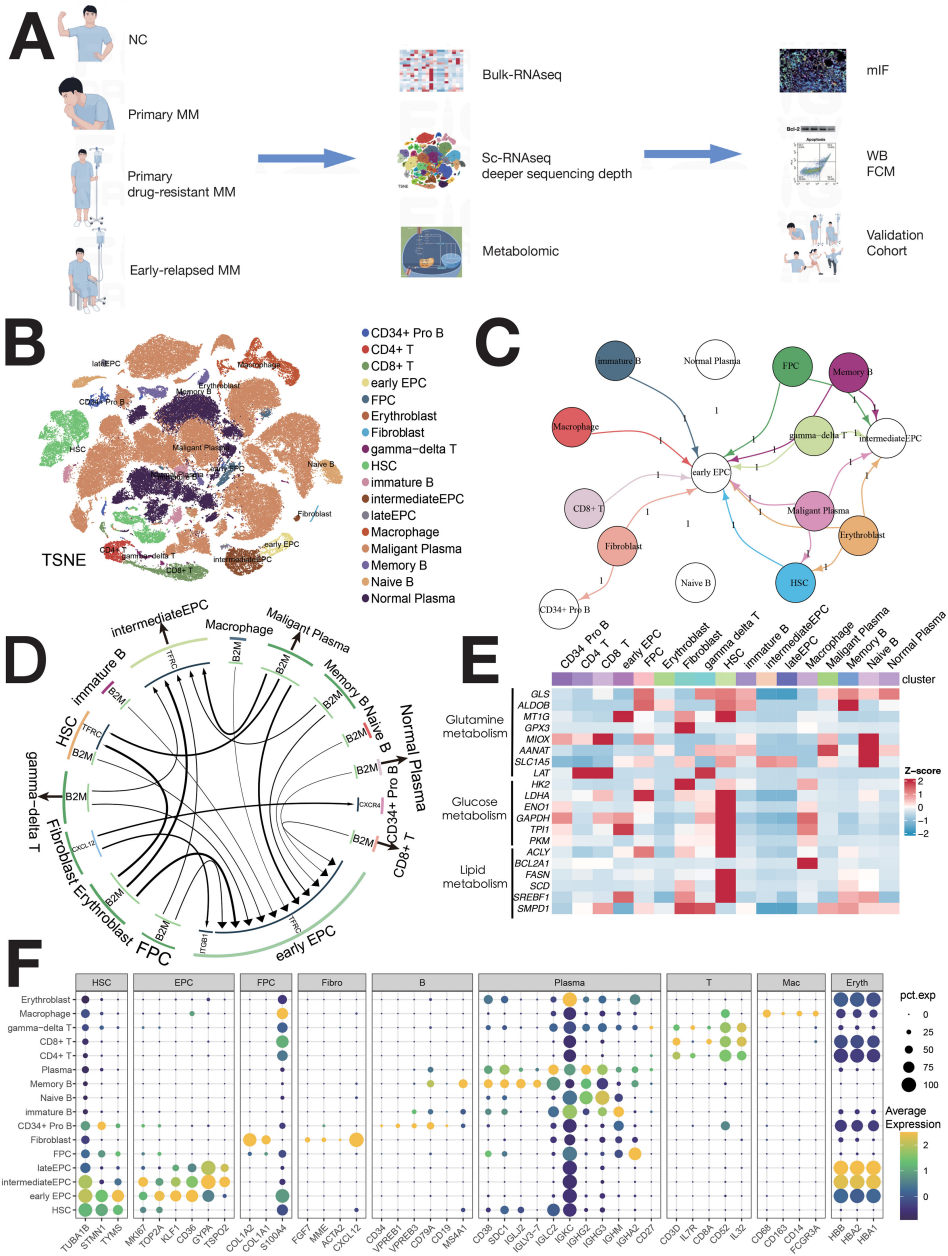
Single-cell sequencing atlas of MM. **(A)** Study workflow. **(B)** MM single-cell sequencing atlas. **(C)** Cellular interactions in the TME of MM. **(D)** Overview of ligand–receptor interactions. **(E)** Enzyme expression heatmap for major metabolic pathways. **(F)** Normalized expression of cell type-specific genes in 16 MM populations.

A total of 113 participants were included (**Figure 1A**), with 111,640 cells identified (82,078 PCs, 29,562 non-PCs). Cell clusters were annotated using canonical markers (4–6), including pro-B, CD4+ T, CD8+ T, γδ T, immature B, macrophages, memory B, naïve B, PCs, EPCs (early/intermediate/late), fibroblasts, and HSCs (**Figure 1B**). There are marked differences in enzymatic activity of distinct metabolic pathways between cell types (**Figure 1E**). Bubble maps visualized clusters (**Figure 1F**). Cell-cell communication was analyzed using iTALK, revealing ligand-receptor pairs in the TME (**Figures 1C, D**).

#### 3.1.1 HSCs

**Figure 2:**
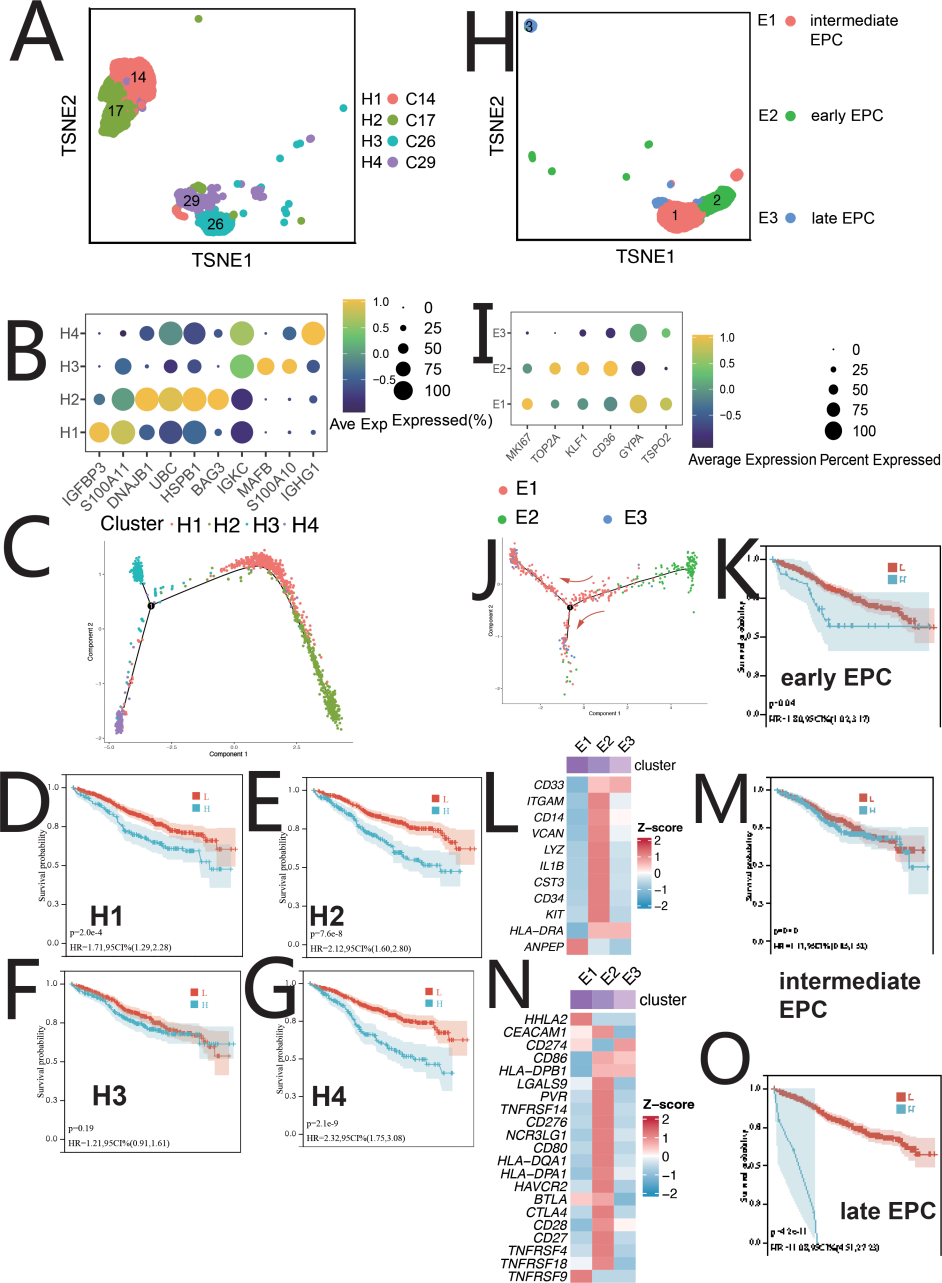
Single-cell sequencing profile of HSCs and EPCs. **(A)** tSNE for HSCs. **(B)** Gene expression dot plot for HSC clusters. **(C)** Pseudo-time analysis of HSC clusters. **(D)** Correlation between H1 abundance and overall survival (OS) using CIBERSORTx. **(E)** Correlation between H2 abundance and OS using CIBERSORTx. **(F)** Correlation between H3 abundance and OS using CIBERSORTx. **(G)** Correlation between H4 abundance and OS using CIBERSORTx. **(H)** tSNE for EPCs. **(I)** Gene expression dot plot for EPC clusters. **(J)** Pseudo-time analysis of EPC clusters. **(K)** Correlation between early EPC abundance and OS using CIBERSORTx. **(L)** Expression of myeloid-related genes in EPC clusters. **(M)** Correlation between intermediate EPC abundance and OS using CIBERSORTx. **(N)** Immune checkpoint expression in EPC clusters. **(O)** Correlation between late EPC abundance and OS using CIBERSORTx.

HSCs were classified into four clusters: H1 (IGFBP3+S100A11+), H2 (BAG3+DNAJB1+), H3 (MAFB+S100A10+), and H4 (IGHG1+IGKC+) (**Figures 2A, B**). Pseudo-time analysis (**Figure 2C**) revealed two differentiation pathways: H4/H3 and H4/H1/H2. H4 upregulated IFN-α, inflammation, and angiogenesis, while H1/H2 showed MYC, mTOR, and NOTCH signaling (**Supplementary Figure S2B**). Distinct TF regulation was observed (**Supplementary Figure S2C**). Survival analysis showed a negative correlation between H1/H2/H4 abundance and patient survival (**Figures 2D–G**). H1/H2/H4 were involved in tumor-immune crosstalk, unlike H3 (**Supplementary Figures S10A, B**).

#### 3.1.2 EPCs

EPCs were divided into three clusters: early EPCs (E2, TSPO2+), intermediate EPCs (E1, CD36+), and late EPCs (E3, IFIT1B+) (**Figures 2H, I**, **Supplementary S2D**). Pseudo-time analysis (**Figure 2J**) showed E2, E1, and E3 in early, mid, and late stages. E2 cells expressed myeloid markers and immune checkpoints (**Figures 2L, N**) and were central in cell communication (**Figures 1C**, **Supplementary Figure S10A**). E2 cells, termed MEPCs, showed upregulated MYC and angiogenic pathways with enhanced glycolysis. E1/E3 were regulated by GATA1/RUNX2, while E2 was regulated by MYC, NR2C2, and USF1. Survival analysis showed correlation between EPCs abundance and survival (**Figures 2K, M, O**). MEPCs were confirmed by mIF staining (**Supplementary Figure S10C**) and were more abundant during relapse (**Supplementary Figure S10J**).

#### 3.1.3 Erythroblasts

**Figure 3:**
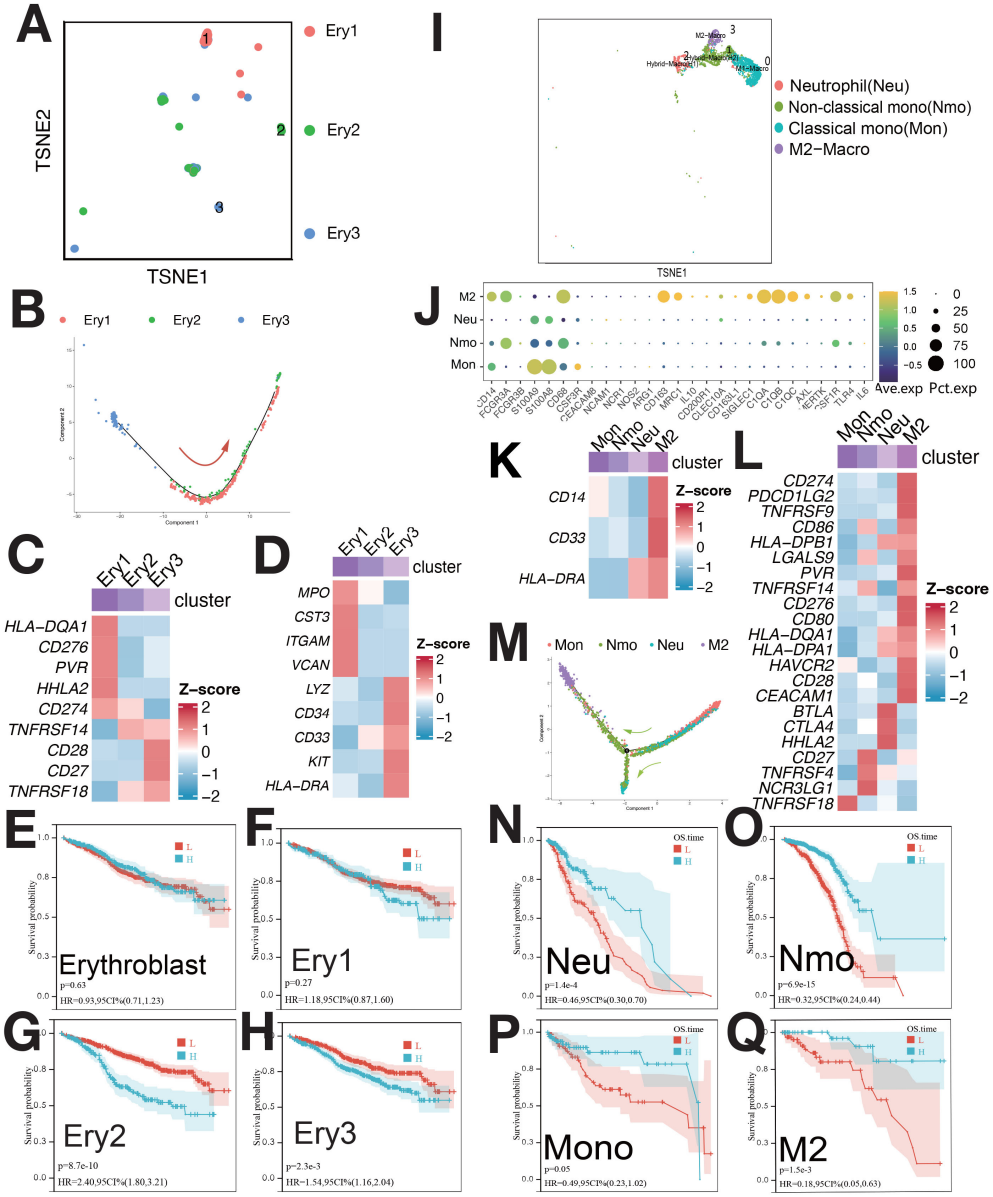
Single-cell sequencing profile of erythroblasts and macrophages. **(A)** tSNE for erythroblasts. **(B)** Pseudo-time analysis of erythroblast clusters. **(C)** Expression of immune checkpoints in erythroblast clusters. **(D)** Expression of myeloid-related genes in erythroblast clusters. **(E)** Correlation between erythroblast abundance and OS using CIBERSORTx. **(F)** Correlation between Ery1 abundance and OS using CIBERSORTx. **(G)** Correlation between Ery2 abundance and OS using CIBERSORTx. **(H)** Correlation between Ery3 abundance and OS using CIBERSORTx. **(I)** tSNE for myeloid. **(J)** Gene expression dot plot for myeloid clusters. **(K)** Expression of myeloid-related genes in myeloid clusters. **(L)** Expression of immune checkpoints in myeloid clusters. **(M)** Pseudo-time analysis of myeloid clusters. **(N)** Correlation between Neu abundance and OS using CIBERSORTx. **(O)** Correlation between Nmo abundance and OS using CIBERSORTx. **(P)** Correlation between Mono abundance and OS using CIBERSORTx. **(Q)** Correlation between M2 abundance and OS using CIBERSORTx.

Erythroblasts were classified into Ery1 (MPO+), Ery2 (CCND1+), and Ery3 (LYZ+) (**Figures 3A**, **Supplementary Figure S3A**). Pseudo-time analysis (**Figure 3B**) showed Ery3, Ery1, and Ery2 in early, middle, and late stages. Ery1 and Ery3 expressed myeloid markers and immune checkpoint genes (**Figures 3C, D**) (7). Ery3 showed upregulated angiogenesis (**Supplementary Figure S3B**). Ery2 abundance correlated with poorer survival, while Ery3 was linked to better outcomes (**Figures 3G, H**). The survival analysis of Erythroblast and Ery1 showed no statistical significance (**Figures 3E, F**).

#### 3.1.4 Myeloid

Myeloid were categorized into Ma0/Mon (CD14+FCGR3A±), Ma1/Nmo (FCGR3A+CD14low+C1QA+C1QB), Ma2/Neu (S100A8+S100A9+CEACAM8+), and Ma3/M2 (CD68+CD163+)(**Figures 3I, J**). M2 cells had high CD14, CD33, and low HLA-DRA expression (**Figure 3K**) also, as MDSC [myeloid-derived suppressor cells (MDSCs)], and overexpressed immune checkpoints (**Figure 3L**). These cells correlated with survival (**Figure 3Q**). mIF confirmed M2/MDSC cells (**Supplementary Figures S10D, E**), with lower abundance during relapse compared to diagnosis (**Supplementary Figure S10J**).

#### 3.1.5 CD8+ T cells, CD4 T cells, and γδ T cells

**Figure 4:**
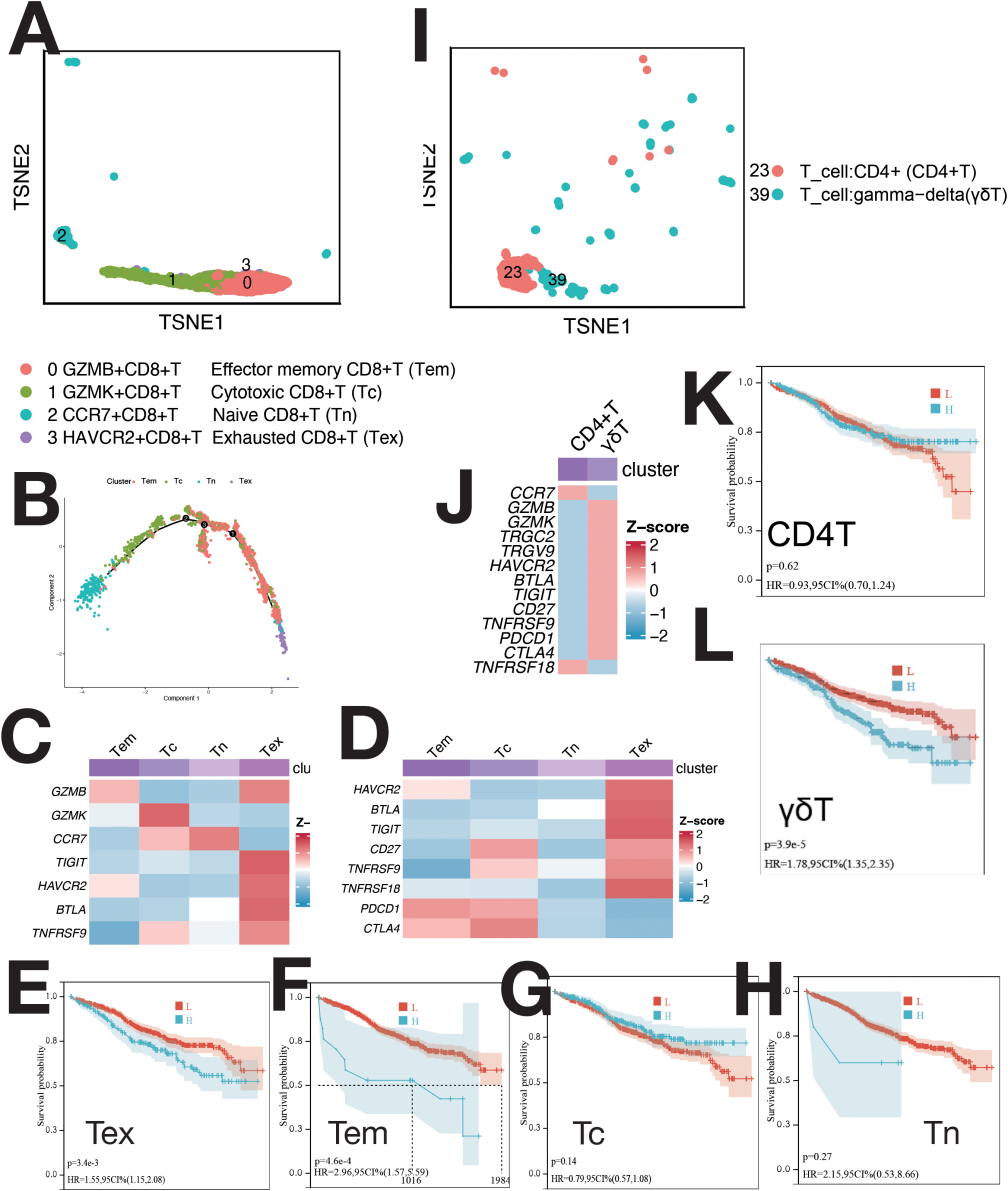
Single-cell sequencing profile of T cells. **(A)** tSNE for CD8+ T cells. **(B)** Pseudo-time analysis of CD8+ T cell clusters. **(C)** Gene expression heatmap for CD8+ T cells. **(D)** Expression of immune checkpoints in CD8+ T cells. **(E)** Correlation between Tex abundance and OS using CIBERSORTx. **(F)** Correlation between Tem abundance and OS using CIBERSORTx. **(G)** Correlation between Tc abundance and OS using CIBERSORTx. **(H)** Correlation between Tn abundance and OS using CIBERSORTx. **(I)** tSNE for CD4+ T and γδ T cells. **(J)** Expression of markers and immune checkpoints in CD4+ and γδ T cells. **(K)** Correlation between CD4+ T cell abundance and OS using CIBERSORTx. **(L)** Correlation between γδ T cell abundance and OS using CIBERSORTx.

CD8+ T cells were divided into four clusters: GZMB+ (Tem), GZMK+ (Tc), CCR7+ (Tn), and HAVCR2+ (Tex) (**Figures 4A, C**). Pseudo-time analysis (**Figure 4B**) showed HAVCR2+ CD8+ T cells as exhausted (Tex), expressing HAVCR2, BTLA, and TIGIT (8). CCR7+ T cells were naive (Tn) (8), while GZMB+ and GZMK+ T cells were Tem and Tc (8). Tem and Tc expressed PDCD1 and CTLA4; Tex cells expressed HAVCR2, BTLA, TIGIT, and CD27 (**Figure 4D**). CD4 T cells and γδ T cells were not further classified due to low proportions (**Figure 4I**). CD4 T cells expressed CCR7; γδ T cells expressed TRGC2, TRGV9, GZMK, and GZMB (**Supplementary Figure S4D**). γδ T cells showed elevated expression of immunosuppressive checkpoints (**Figure 4J**). The abundance of Tex, Tem, and γδ T cells negatively correlated with survival (**Figures 4F, G, L**). mIF staining confirmed Tex and γδ T cells (**Supplementary Figures S10F, G**), with increased levels during relapse (**Supplementary Figure S10J**).

#### 3.1.6 B cells (excluding plasma cells)

**Figure 5:**
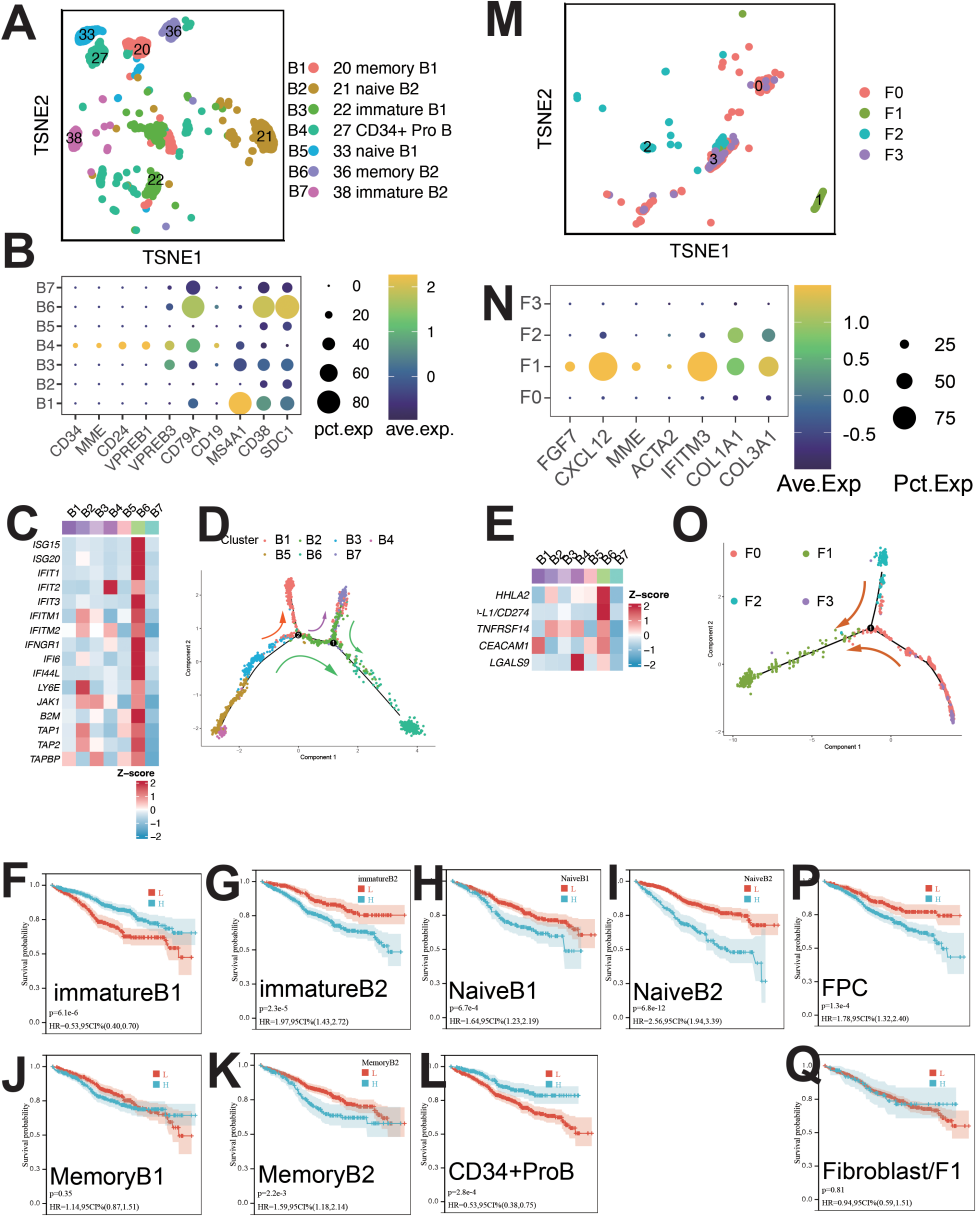
Single-cell sequencing profile of B cells and fibroblasts. **(A)** tSNE for B cells. **(B)** Gene expression dot plot for B cell clusters. **(C)** Expression of interferon-stimulated and antigen-presenting genes in B cells. **(D)** Pseudo-time analysis of B cell clusters. **(E)** Expression of immune checkpoints in B cells. **(F)** Correlation between immature B1 abundance and OS using CIBERSORTx. **(G)** Correlation between immature B2 abundance and OS using CIBERSORTx. **(H)** Correlation between naïve B1 abundance and OS using CIBERSORTx. **(I)** Correlation between naïve B2 abundance and OS using CIBERSORTx. **(J)** Correlation between memory B1 abundance and OS using CIBERSORTx. **(K)** Correlation between memory B2 abundance and OS using CIBERSORTx. **(L)** Correlation between CD34+ ProB abundance and OS using CIBERSORTx. **(M)** tSNE for fibroblasts. **(N)** Gene expression dot plot for fibroblast clusters. **(O)** Pseudo-time analysis of fibroblast clusters. **(P)** Correlation between FPC abundance and OS using CIBERSORTx. **(Q)** Correlation between fibroblast abundance and OS using CIBERSORTx.

B cells were classified into seven clusters (**Figure 5A**): B1 (IGLJ3+), B2 (WFDC2+), B3 (EDNRB+), B4 (HIST1H4C+), B5 (MYCL+), B6 (ISG15+), and B7 (CCL3+) (**Supplementary Figure S5A**). B4 cells were CD34+ pro-B cells, B2 and B5 as naive B cells, B1 and B6 as memory B cells, and B3 and B7 as immature B cells (**Figure 5B**). Pseudo-time analysis (**Figure 5D**) showed B4 in early differentiation, with B1 and B6 at opposite ends.

B6 cells upregulated pathways linked to IFN-α, IFN-γ, protein secretion, UPR, P53, PI3K/AKT/MTOR, EMT, angiogenesis, and TNFA signaling (**Supplementary Figure S5B**). High expression of ISGs, antigen-presenting genes (B2M, TAP1), and immune checkpoints (PD-L1, HHLA2, TNFRSF14, CEACAM1, LGALS9) was observed in B6 cells (**Figure 5C**). B6 cells were regulated by IRF9 and STAT2 (**Supplementary Figure S5C**).

Survival analysis showed positive correlation with B1 and B4 cells, and negative with B7, B6, B5, and B2 cells (**Figures 5F–L**). mIF staining confirmed ISG15+ B cells (**Supplementary Figure S10H**), with elevated levels during relapse and initial diagnosis (**Supplementary Figure S10J**).

#### 3.1.7 Plasma cells

**Figure 6:**
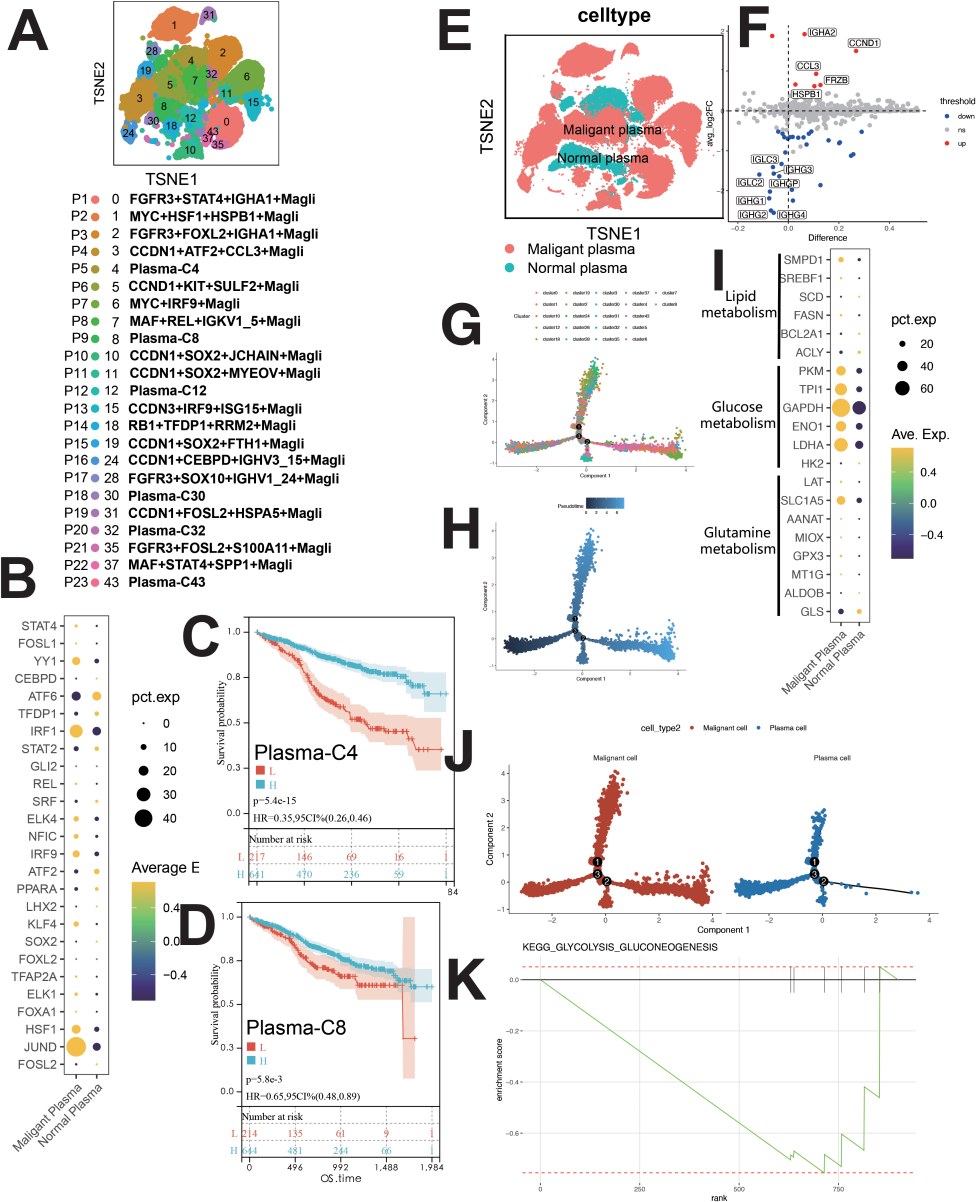
Single-cell sequencing profile of MM cells (including MMCs and NCs). **(A)** tSNE for MM cells. **(B)** Upregulated and downregulated TFs of malignant and normal plasma. **(C)** Correlation between Plasma-C4 abundance and OS using CIBERSORTx. **(D)** Correlation between Plasma-C8 abundance and OS using CIBERSORTx. **(E)** tSNE for normal and malignant PCs. **(F)** Differential gene expression between normal and malignant PCs. **(G)** Pseudo-time analysis of MM cells by cluster. **(H)** Pseudo-time analysis of MM cells by pseudotime. **(I)** Gene expression dot plot for enzymes related to major metabolic pathways. **(J)** Higher glycolytic activity in malignant PCs than in normal PCs. **(K)** Enrichment plot for KEGG glycolysis/gluconeogenesis pathway, this figure shows the normal plasma cells.

PCs were categorized into 23 clusters (**Figure 6A**), each with unique gene, TF, mutation, and immunophenotypic profiles (**Supplementary Figures S6A, C, E**). Clusters were named based on mutations, TFs, and signature genes (**Figure 6A**). Normal plasma cells (NCs) and MM cells (MMCs) were differentiated by (1): MMC-specific mutations (MYC, CCND1/CCND3, FGFR3/MMSET, MAF/MAFB) (9, 10) (2); karyotypic changes (17p-, 13q14-, t (11, 12) /IgH-FGFR3, t (8, 12) /IgH-CCND1, etc.) (12, 13) (3); immunophenotype (CD38dimCD138+CD19-CD20+CD56+CD28+CD27- and CD38+CD138+CD19+CD20-CD56-CD28-CD27+ NCs) (14).

Clusters P1-P4, P6-P8, P10-P17, P19, P21, P22 (malignant PCs) and P5, P9, P12, P18, P20, P23 (NCs) were identified (**Figures 6A**, **Supplementary Figure S6C**). MMCs showed high CCND1 and CCL3 expression, while NCs expressed IGHG1 and IGHG2 (**Figures 6E, F**).

Malignant PCs exhibited high JUND, IRF1, and YY1 expression, while NCs showed elevated ATF6 (**Figure 6B**). Pseudo-time analysis suggested similar development for normal and malignant PCs (**Figures 6G, H, J**). GSVA revealed downregulation of apoptosis in MMCs, with upregulation in OXPHOS, angiogenesis, MYC signaling, E2F, DNA repair, fatty acid metabolism, bile acid metabolism, and glycolysis (**Supplementary Figure S6D**). GSEA confirmed glycolysis upregulation in MMCs (**Figure 6K**), supported by metabolic analysis (**Supplementary Figure S1B**).

Survival analysis revealed positive correlations between Plasma-C4, Plasma-C8, CCND1+KIT+SULF2+ Magli, and MAF+REL+IGKV1_5+ Magli cells and patient survival (**Figures 6C, D**, **Supplementary Figure S7M, R**). Negative correlations were observed between various malignant plasma cell subpopulations (e.g., RB1+TFDP1+RRM2+ Magli, CCDN3+IRF9+ISG15+ Magli, etc.) and patient survival (**Supplementary Figures S7D–S**).

#### 3.1.8 stromal cells

Fibroblasts and fibroblast progenitor cells (FPCs) were classified into four clusters (**Figure 5M**): F0 (LAMP5+), F1 (CXCL12+), F2 (BIRC3+), and F3 (CCL3+) (**Supplementary Figure S5D**). F1 cells expressed CAF-related markers (COL1A1, COL3A1, CXCL12, MMP2) (**Figures 5N**, **Supplementary Figure S5D**) and were identified as CAFs (15). Pseudo-time analysis (**Figure 5O**) showed F0, F1, F2 and F3 in early, mid, and late stages. Upregulated pathways in F1 cells included IL6/JAK/STAT3 and IFN-γ signaling (**Supplementary Figure S5E**). Survival analysis indicated a negative correlation between FPC abundance and patient survival (**Figure 5P**). CAF presence was confirmed by mIF staining (**Supplementary Figure S10I**), with increased abundance during relapse compared to initial diagnosis (**Supplementary Figure S10J**).

#### 3.1.9 Survival analysis

CIBERSORTx evaluated the abundance of cell clusters in the MMRF cohort (bulk RNA-seq) using deconvolution. Kaplan-Meier (KM) analysis examined the correlation between cell cluster abundance and patient survival (**Figures 2D–G, K, M, O**, **3E–H, N–Q**, **4E–H, K, L**, **5F–L, P–Q**, **6C, D**, **Supplementary Figures S7A–T**). A negative correlation (P<0.05) was found between survival and the abundance of immature B2 cells, memory B1, naive B1/B2 cells, CD8+ Tem/Tex cells, late/early EPCs, γδ T cells, HSC-C29/C17/C14 cells, FPCs, Ery2 cells, and multiple Magli cell clusters (**Supplementary Figures S7D–S**). A positive correlation (P<0.05) was found between survival and the abundance of CD34+ pro-B cells, immature B1 cells, Hy1/Hy2 cells, M1/M2 macrophages, Ery3 cells, C4/C8 PCs, and several Magli cell clusters.

### 3.2 Enhanced IFN-γ and IFN-α signaling

Cell-cell communication analysis (**Supplementary Figure S10A**) identified four major cell types: hematopoietic stem cells, B cells, tumor cells, and erythroid cells. Among these populations, we identified significantly upregulated pathways: KRAS, IFN-γ, IFN-α, IL6-JAK-STAT3, PI3K-AKT-MTOR and MYC targets (**Supplementary Figure S8A**). Notably, IFN-γ and IFN-α signaling pathways were particularly upregulated— a novel finding that will be discussed in detail later (Section 3.2).

### 3.3 Validation cohort

Retrospective analysis showed elevated levels of IFN-γ, IL-6, CRP, β-2M, and ferritin in MM patients compared to controls. Serum Fe, TF, and TSAT were reduced in the myeloma cohort (**Supplementary Figure S8G**).

### 3.4 Sc-metabolism profiling analysis

A comparison of five major metabolic pathways in individual cells is shown in **Supplementary Figure S1B**. Malignant PCs exhibited elevated glycolysis compared to normal PCs (**Supplementary Figure S1B**). GSEA confirmed glycolytic pathway upregulation in MMCs (**Figure 6K**). Enzymes involved in glycolysis and glutamine metabolism (LDHA, ENO1, GAPDH, TPI1, PKM, SLC1A5) were higher in malignant PCs (**Figure 6I**). The OXPHOS pathway was upregulated in malignant PCs (**Supplementary Figure S6D**).

### 3.5 *In vitro* drug screening

As shown by scMetabolism (**Supplementary Figure S1B**), GSVA (**Supplementary Figure S6D**), and GSEA (**Figure 6K**), glycolysis was elevated in malignant plasma cells (PCs) vs normal PCs. Enzymes involved in glycolysis and glutamine metabolism were upregulated in malignant PCs (**Figure 6I**), and the OXPHOS pathway was also elevated (**Supplementary Figure S6D**).

We selected galloflavin (LDHA inhibitor), CB839 (GLS1 inhibitor), NAC (OXPHOS inhibitor), and EGCG (multi-target inhibitor of glycolysis, glutaminolysis/GDH, and OXPHOS). Meanwhile, two combination regimens targeting metabolic reprogramming were designed: CB839 + EGCG and galloflavin + NAC. CCK8 assays showed significant inhibition of KM3 cell proliferation by these compounds (P<0.05) (**Supplementary Figures S9A–C**). For drug combinations, galloflavin (20–100 μmol/L) was paired with NAC (2–10 μmol/L), and CB839 (1.25–5 μmol/L) was combined with EGCG (40–120 μmol/L). CCK8 assays assessed effects on cell proliferation. Galloflavin-NAC combinations had no CI <1, indicating no synergy. Among CB839-EGCG combinations, the CB839 (5 μmol/L) + EGCG (120 μmol/L) combination had the lowest CI, suggesting the strongest synergy. This combination was selected for subsequent experiments (**Supplementary Figure S9D**). Flow cytometry confirmed that the G2-phase proportion significantly increased (P<0.05), and the G1-phase proportion decreased (P<0.05) in the combined treatment group vs controls. Apoptotic rate was also higher in the combined treatment group (P<0.05) (**Supplementary Figures S9E–J**). Western blotting showed lower Bax (P<0.05) and higher Bcl-2 (P<0.05) expression in the blank group compared to the combined treatment group. The Bax/Bcl-2 ratio was higher in the combined treatment group (P<0.05) (**Supplementary Figures S9E, F**).

## 4 Discussion

Multi-omics, including single-cell transcriptomics and sc-Metabolism, overcome bulk sequencing limitations, enabling precise characterization of pathogenesis and metabolic reprogramming. Cell–cell communication analysis identified critical pathways (IFN-γ/IFN-α signaling, B2M-TFRC), leading to a novel MM pathogenesis model. We also explored single-cell metabolic reprogramming and screened for therapeutic agents.

### 4.1 Disrupted TME

In this study, we identified several key features of hematopoietic stem cells (HSCs), endothelial progenitor cells (EPCs), erythrocytes, macrophages, lymphocytes, and malignant plasma cells (PCs) within the multiple myeloma (MM) tumor microenvironment (TME). Some of these have not been mentioned in previous studies and are worthy of further research in the future. HSCs, particularly MAHSCs (IGHG1+, BAG3+, IGFBP3+), play a crucial role in MM pathogenesis, with a negative correlation between their abundance and patient survival (**Figures 2G, E, D**). Cell–cell communication analysis further revealed that these HSCs upregulated cell proliferation and angiogenesis, with significant pathways such as IL2/STAT5, IL6/JAK/STAT3, IFN-α, and IFN-γ in H4 cells (**Supplementary Figure S3B**). EPCs, especially early EPCs (E2), were critical in the TME, showing high expression of myeloid markers and immune checkpoints, and correlating with poor prognosis (**Figures 1C**, **2N**, **Supplementary Figure S10B**). Survival analysis showed that EPCs, including intermediate and late EPCs, play a central role in immunosuppression, with high expression of cancer-driving genes and immune checkpoint ligands (**Figure 2N**, **Supplementary Figure S2F**). Erythroblasts (Ery1 and Ery2) mediated immunosuppression, whereas Ery3 promoted immune-mediated tumor killing, with survival analysis revealing positive correlations for Ery3 abundance (**Figures 3F–H**). Macrophages, including the M2 subset and MDSCs, showed upregulation of immunosuppressive markers (**Figures 3J, 3L**) (16–18), consistent with the study by Favaloro J (17). M2 macrophages expressed both co-stimulatory and immunosuppressive molecules (e.g., CD28 and CD274), suggesting an advantage for the CD28 pathway, while MDSCs also played a significant role in immunosuppression and were confirmed by mIF staining (**Supplementary Figures S10D, E**). T lymphocytes, including CD4+ and γδ T cells, exhibited exhaustion markers such as PD-1 and CTLA4, limiting their tumor-killing capacity, with CIBERSORTx showing no correlation between T cell subsets and survival (**Figures 4E–L**). The expression of immune checkpoints such as PD-1 and HAVCR2 across T cell subsets suggests these as combined targets for MM immunotherapy (**Figures 4D, J**), similar to the study by Lv J (19). ISG15+ B cells, which differentiate from B cells, showed upregulation of pathways related to protein secretion and immune suppression and correlated with poor prognosis (**Supplementary Figures S5B**, **5C, K**) (20). Their elevated expression of PD-L1 and enrichment in interferon genes further supports their role in immune evasion (**Figures 5C, E**). These viewpoints have been confirmed by the studies of Dubrot J and Zhao R (21, 22). Malignant PCs exhibited substantial genetic heterogeneity, with mutations in cyclin D1, cyclin D2/D3, and MAF, contributing to abnormal gene expression patterns and correlating with poor survival outcomes (**Supplementary Figures S7D–S**). Finally, cancer-associated fibroblasts (CAFs), identified by their high expression of CAF markers, interacted with EPCs and CD34+ Pro-B cells via CXCL12/CXCR4, promoting tumor progression and angiogenesis (**Figures 1D**, **Supplementary Figures S10I, S5D, N**), as found in many tumor studies where CAFs promote tumor invasion via CXCL12/CXCR (23–29). These findings provide a comprehensive view of the TME in MM, highlighting the interactions between various cell types that contribute to disease progression and offering potential therapeutic targets for intervention.

### 4.2 Cellular communication and novel mechanisms in MM

**Figure 7:**
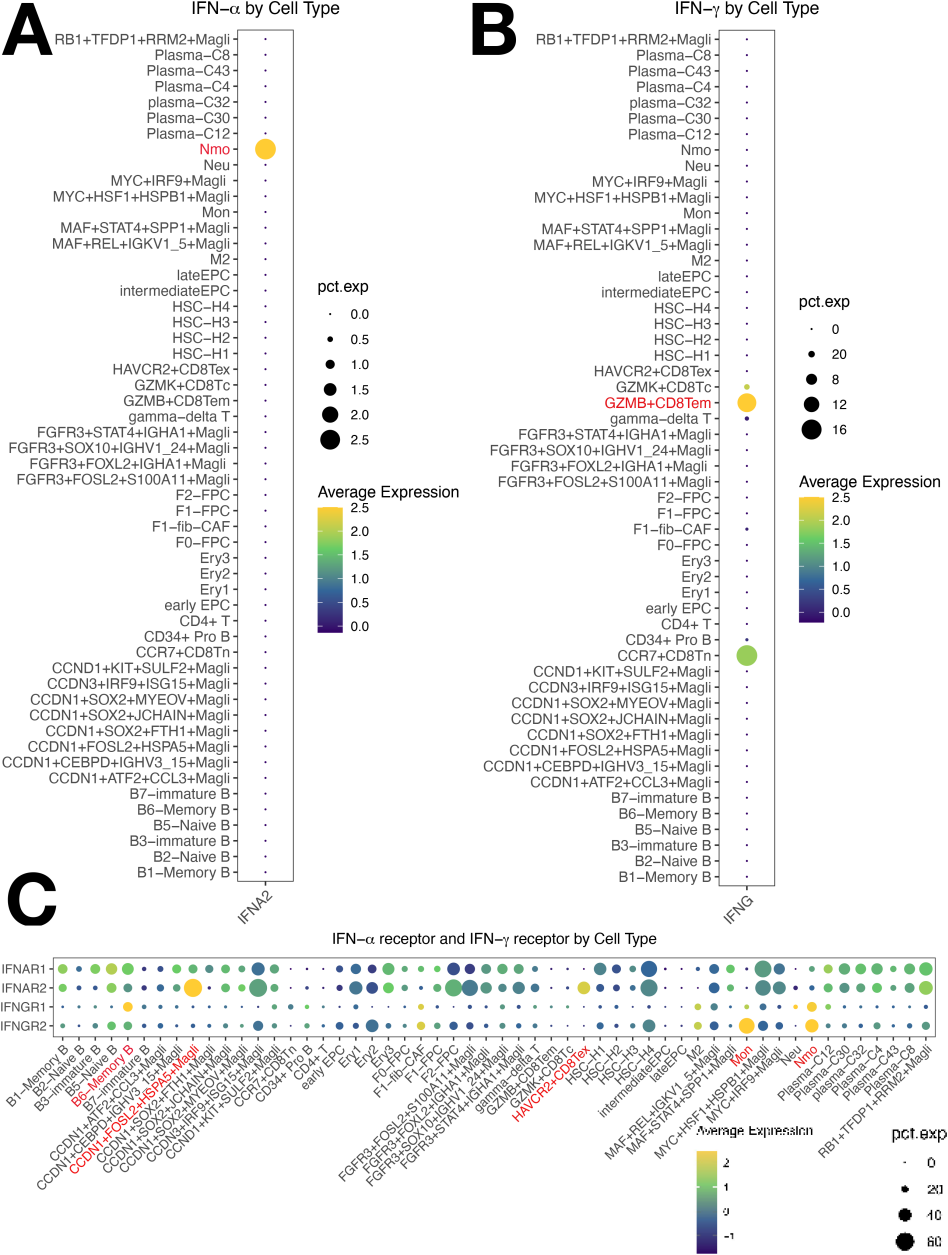
Cellular Expression of IFN-γ/IFN-α Pathways in Multiple Myeloma (MM) Cells. **(A)** IFN-α is predominantly expressed in non-classical monocytes. **(B)** IFN-γ is predominantly expressed in Tem cells. **(C)** Non-classical monocytes show significantly increased expression of both IFNGR1 and IFNGR2, with ISG15+ B cells showing elevated IFNGR1 expression and monocytes showing elevated IFNGR2 expression.

Using iTALK, early EPCs were the central hub in the network, with memory B2 (B6) and MMCs also central (**Supplementary Figure S10A**). EPCs play a pivotal role in MM. Three key ligand-receptor pairs (B2M-TFRC, CXCL12-CXCR4, CXCL12-ITGB1) were identified, with B2M-TFRC being the most significant (**Figure 1D**). This is the first report showing that B2M-TFRC dominates cellular communication in MM TME. Our analysis identified four major cellular subsets and showed activation of both IFN-γ and IFN-α signaling (**Supplementary Figure S8A**). Tem cells expressed IFNG (**Figure 7B**), mediating IFN-γ secretion. Non-classical monocytes and ISG15+ B cells (B6) had increased IFNGR1 expression (**Supplementary Figure S8B**, **Figure 7C**). Non-classical monocytes and monocytes had increased IFNGR2 expression (**Figure 7C**). IFN-γ/IFNGR binding activated JAK1/STAT1 and IRF1, expressed in H4 and ISG15+ MMCs.

**Figure 8:**
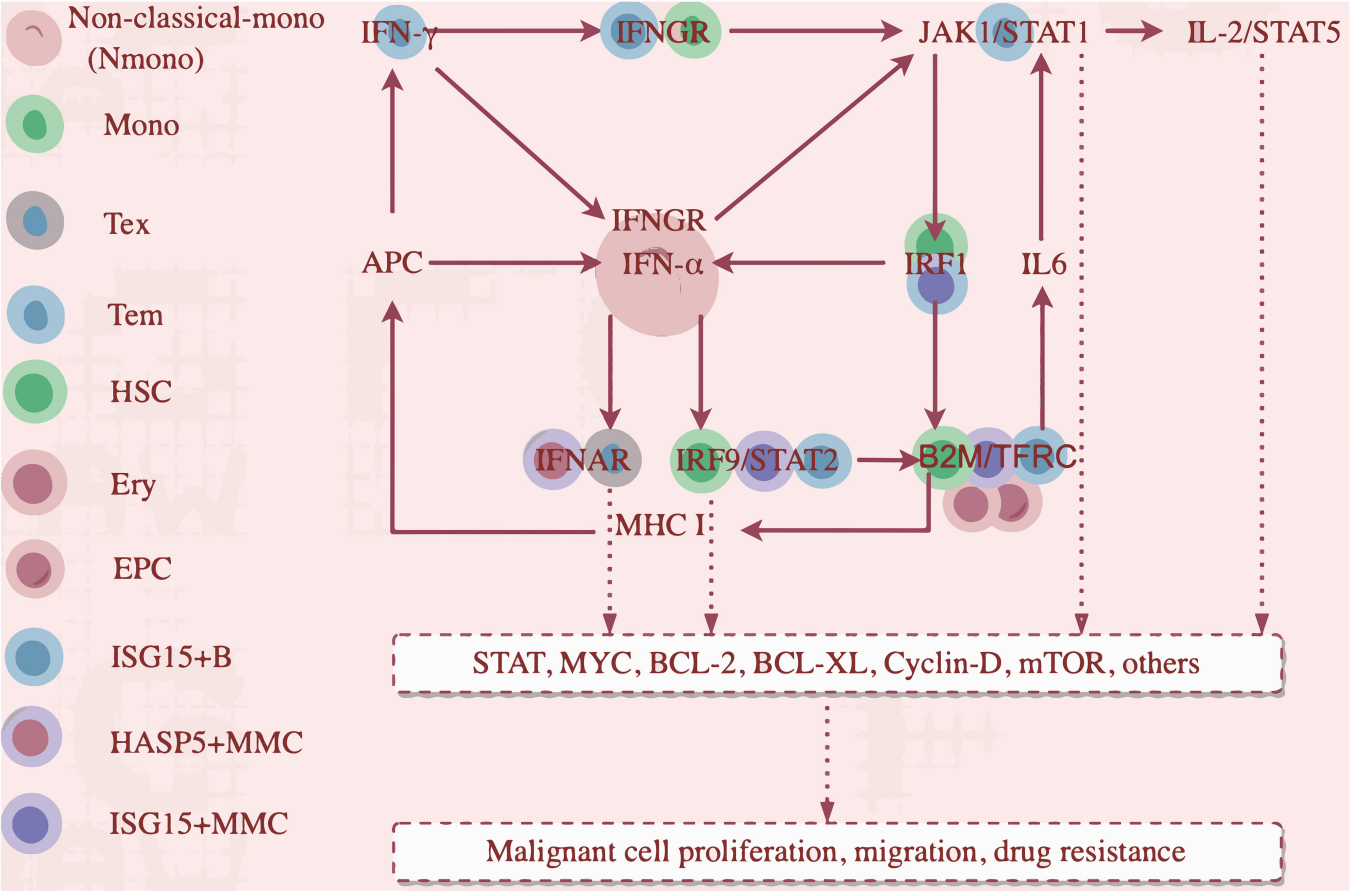
An IFN-g/IFNa-B2M positive feedback loop.

Activated IRF1 drove IFN-α transcription in non-classical monocytes (**Figure 7A**), upregulating IRF9, which complexes with STAT2 to form ISGF3 (IRF9/STAT2). This complex activates B2M expression, an IFN-responsive gene with a super-enhancer (30), enhancing TFR1 (TFRC)expression, activates IL6-STAT3 (31, 32). IRF1 transactivates B2M, part of MHC class I. MHC I upregulates the antigen presentation capacity of Antigen-Presenting Cells (APCs), which in turn stimulates the secretion of IFN-γ and IFN-α. IFN-α also binds with the overexpressed IFNGR2 on Tex and HASP5+ MMCs. Overactivation of IFN-α/IFNAR2 binding promoted the worsening of Tex exhaustion and the resistance of HSPA5+ malignant tumor cells to IFN-α. The overactivated IFN-γ and IFN-α axes converge to drive pathological antigen presentation and reactivation of the pathways, creating a vicious cycle that sustains downstream targets (STAT, MYC, Bcl-2, Bcl-xL), facilitating tumor cell proliferation, migration, and drug resistance. JAK1/STAT1(in T cells) and IL-2/STAT5 are also involved (**Figure 8**).

We propose a cascading reaction model of the IFN-γ/IFN-α-B2M positive feedback loop:

**Primary cascade:**

IFNγ/IFNGR1

(Tem/Nmono,mono,ISG15+B)

↓

IFNα/IFNAR

(Nmono/HSPA5+MMC,Tex)

↓

ISGF3(IRF9/STAT2)

(HSCs,ISG15+B cells,ISG15+MMC).

**Secondary cascade:**

ISGF3(IRF9/STAT2)

(HSCs,ISG15+B cells,ISG15+MMC)

↓

B2M-TFRC

(HSCs,ISG15+B cells,ISG15+MMC,EPCs,erythrocytes)

↓

IL6/JAK/STAT and IFNγ/IFNα.

This pathway highlights IFN-γ/IFN-α signaling and B2M-TFRC in driving MM progression via interferon crosstalk and inflammatory signaling. Our clinical cohort confirmed elevated IFN-γ, IL-6, CRP, β-2M, and ferritin in MM patients, with increased β-2M triggering hepcidin secretion, suppressing TRFC-mediated iron uptake, and reducing TF synthesis. This leads to hypoferremia and decreased TSAT, consistent with reduced serum iron, TF, and TSAT in our cohort (**Supplementary Figure S8G**). And, TFRC and B2M are involved in ferroptosis, linking MM and ferroptosis. Iron chelators inhibit MM cell proliferation *in vitro* (33). Ferroptosis and MM is an emerging research direction.

### 4.3 Targeting metabolic reprogramming in multiple myeloma

Malignant plasma cells exhibited significant metabolic reprogramming, with enhanced glycolysis, OXPHOS activity, and overexpression of glycolytic/glutaminolytic enzymes compared to normal cells (**Supplementary Figures S6D**, **6I, K**, **S1A, B**) (section 2.3). Glucose is metabolized via glycolysis and OXPHOS, the primary ATP pathways.

Metabolic inhibitors used: galloflavin (LDHA), CB-839 (GLS1), NAC (OXPHOS), EGCG (glycolysis, glutaminolysis, OXPHOS).

Both NAC and galloflavin inhibited MM cell proliferation (**Supplementary Figures S9A–C**). LDHA inhibition reduces pyruvate-to-lactate conversion, halting ATP supply (34). Galloflavin non-selectively inhibits LDHA with antitumor activity and low toxicity to normal cells (35). NAC, an antioxidant, showed no significant synergistic effects when combined with galloflavin, possibly due to pharmacological antagonism. Combination inhibits glycolysis/OXPHOS, reducing ATP and triggering negative feedback via mTOR (36, 37).

Glutamine (Gln) is a critical energy substrate for tumor cell proliferation, converted by GLS to α-ketoglutarate for the TCA cycle. MM cells show marked Gln dependence, consuming more Gln and producing more NH_4_^+^ than normal CD138+ cells (38). CB-839, a GLS1 inhibitor, has clinical efficacy in renal carcinoma and refractory tumors (39, 40). EGCG targets glycolytic enzymes (HK, PFK), HIF-1α/GLUT1, and modulates Gln metabolism through GDH inhibition (41–43). The CB-839/EGCG combination inhibited KM3 cell proliferation, with synergistic effects likely mediated through GLS1/GDH, HK/PFK/LDHA, HIF-1α/GLUT1, and OXPHOS blockade.

Tumor cell metabolism is central to oncogenesis and progression (44). Disrupting glycolysis and glutaminolysis impairs protein/DNA synthesis, induces cell cycle arrest, and promotes apoptosis (45). CB-839/EGCG combination induced G2-phase arrest and enhanced apoptosis in KM3 cells, with increased Bax expression and elevated Bax/Bcl-2 ratios, modulating the intrinsic apoptotic pathway.

### 4.4 Limitation

This study has several limitations, including sample heterogeneity, data sparsity, and assumptions inherent to single-cell transcriptomic analysis. Variations in patient samples, such as disease stage and treatment history, may limit the generalizability of our findings. The sparsity of single-cell data may also lead to underrepresentation of rare immune cell populations, and algorithmic assumptions could influence the interpretation of immune cell features. Additionally, the reliance on retrospective data from a single cohort, coupled with the limitations of *in vitro* models, may not fully capture the complexities of the MM microenvironment *in vivo*. While multi-omics integration provides valuable insights, further clinical validation and preclinical studies are necessary to refine and validate these findings.

## 5 Conclusion

Our study generates one of the largest multi-omics MM atlases to date, providing insights into immune evasion mechanisms involving both non-immune and immune cells, newly defined cellular subpopulations, and a potential IFN-γ/IFN-α-B2M feedback loop. Integrated metabolic analysis identified four agents with efficacy against MM, including a superior efficacy combination regimen showing pro-apoptotic and anti-proliferative effects. Above all, this study offers a comprehensive resource for MM progression mechanisms and immunotherapeutic targets.

## Data Availability

The datasets presented in this study can be found in online repositories. The names of the repository/repositories and accession number(s) can be found in the article/**Supplementary Material**.
