# Supplementary figures and images for: Multi-omics reveals immune features in immune and non-immune cells, an IFN-γ/IFN-α-B2M positive feedback loop, and targeted metabolic therapy in multiple myeloma
Source: Front Immunol. 2025 Sep 8;16:1575079. doi: 10.3389/fimmu.2025.1575079 (PMC12450957; doi:10.3389/fimmu.2025.1575079)

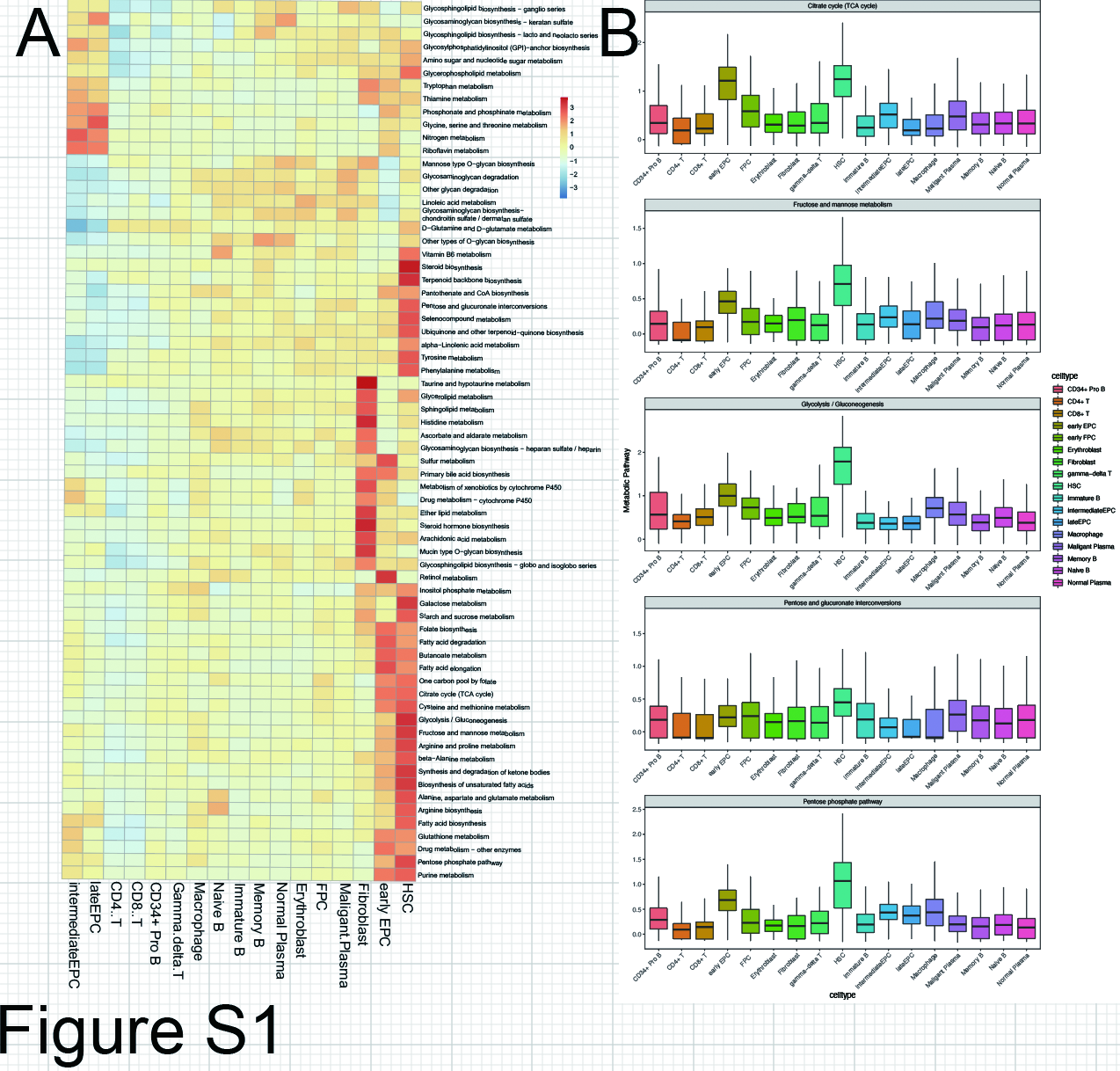

Supplement: Supplementary file 1 [file Image1.tif]

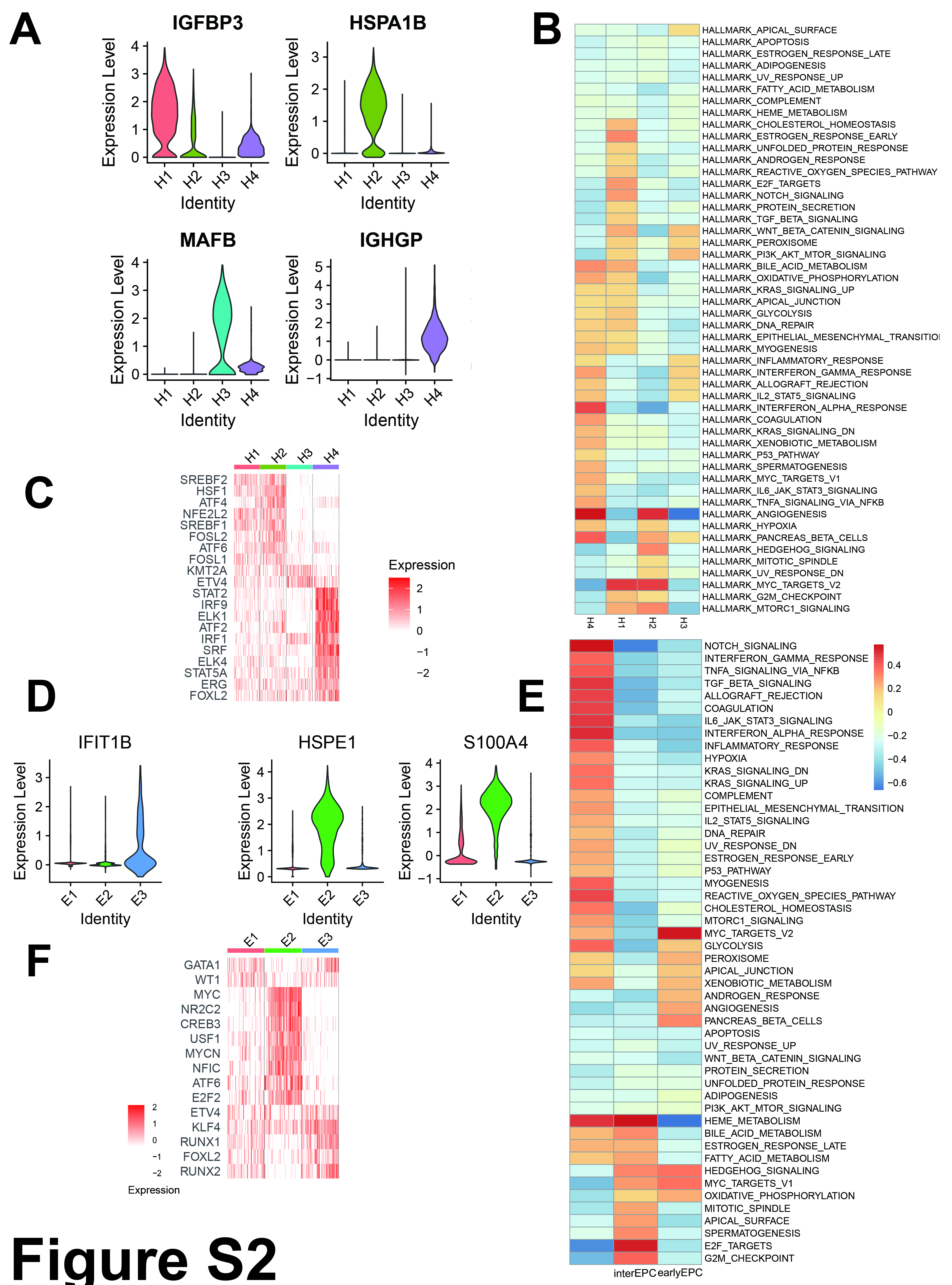

Supplement: Supplementary file 2 [file Image2.tif]

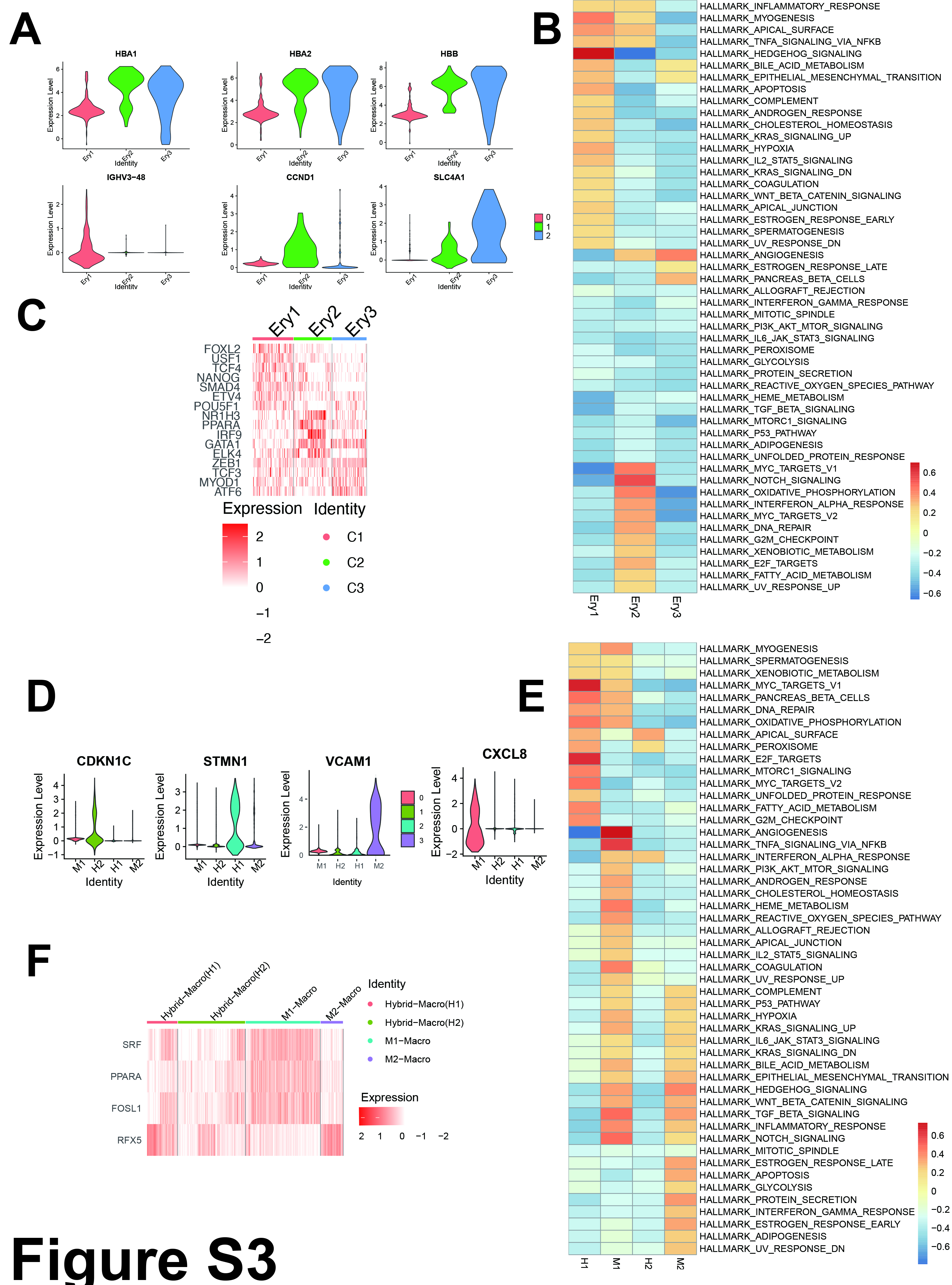

Supplement: Supplementary file 3 [file Image3.tif]

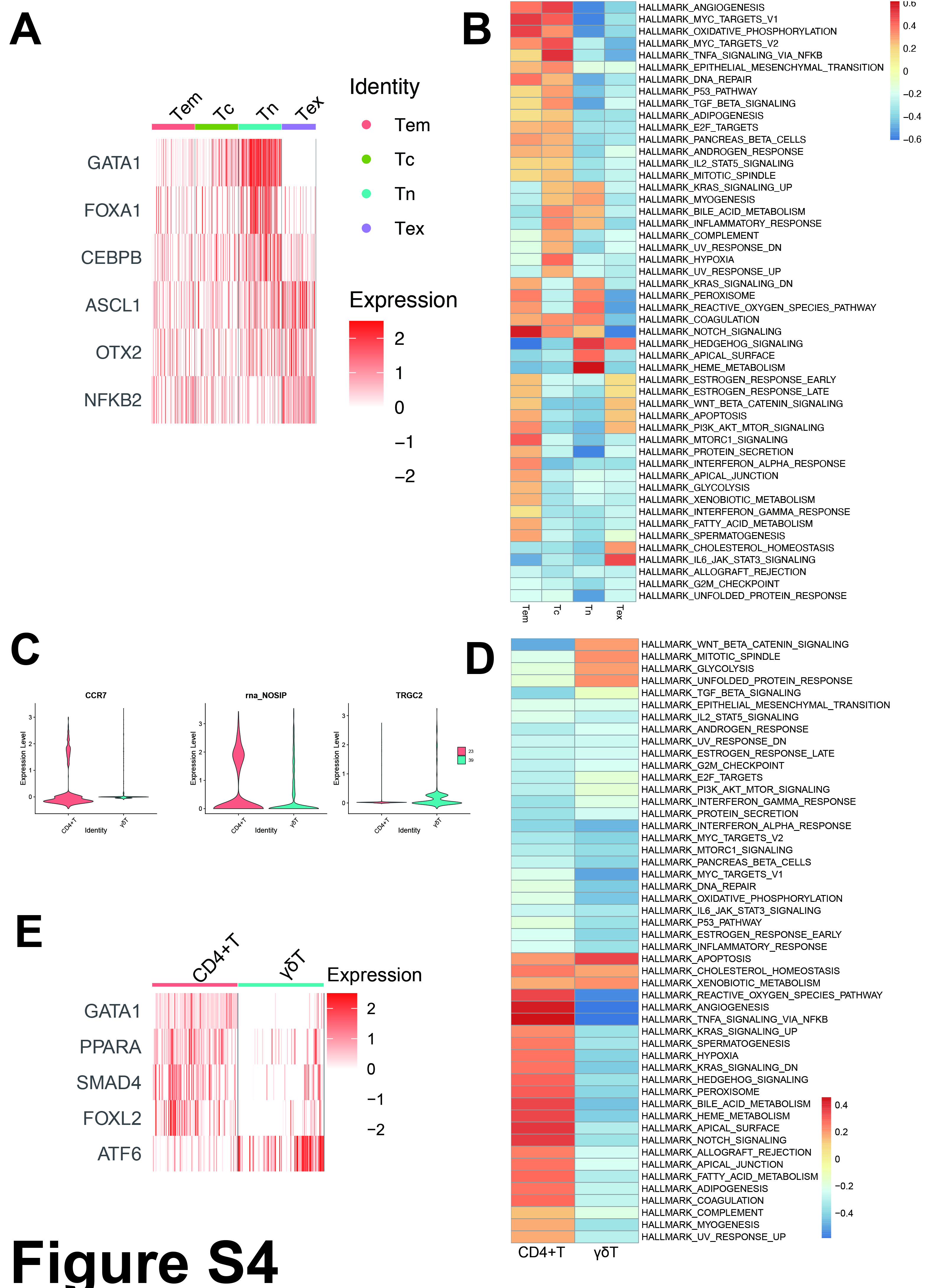

Supplement: Supplementary file 4 [file Image4.tif]

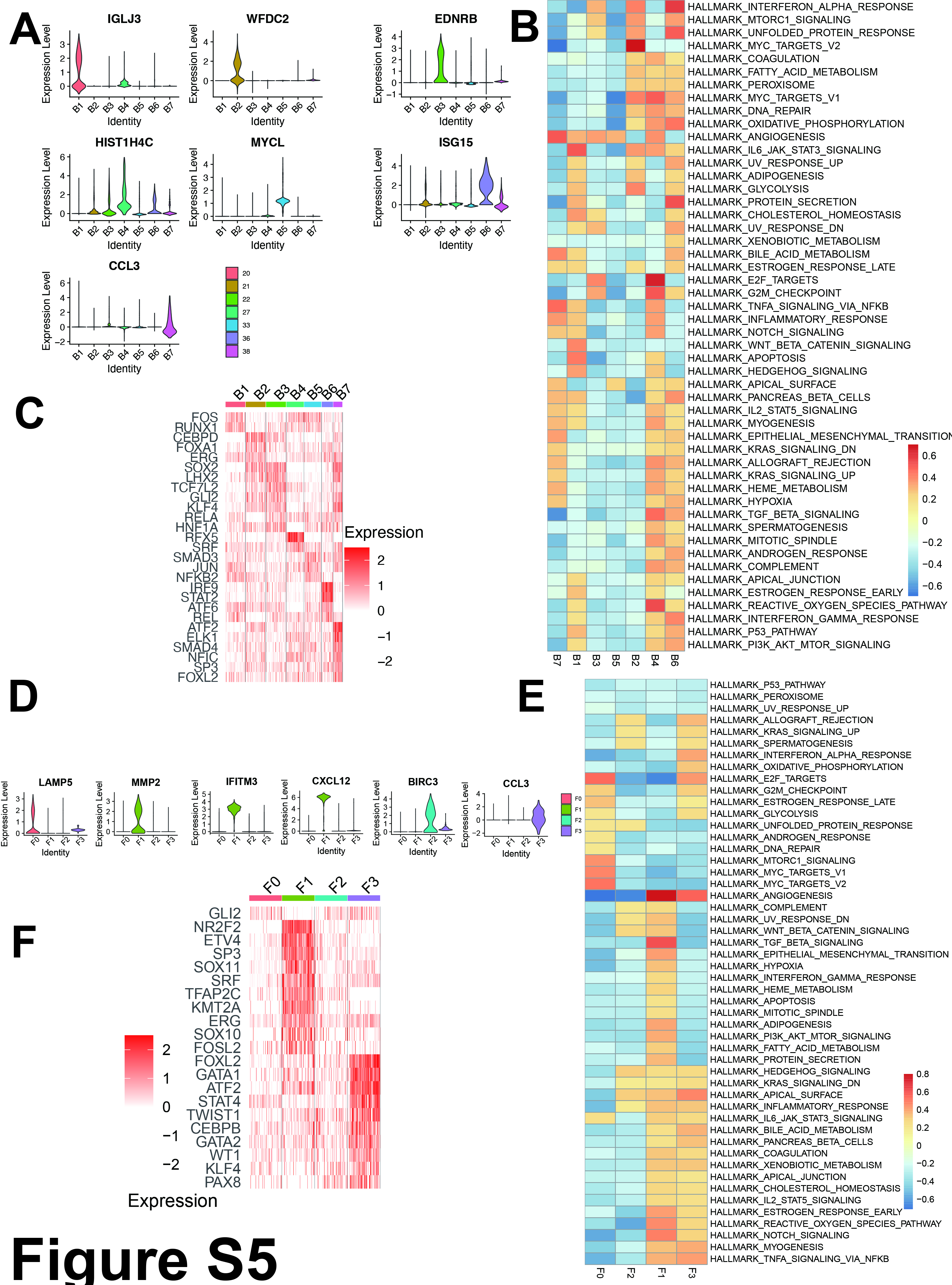

Supplement: Supplementary file 5 [file Image5.tif]

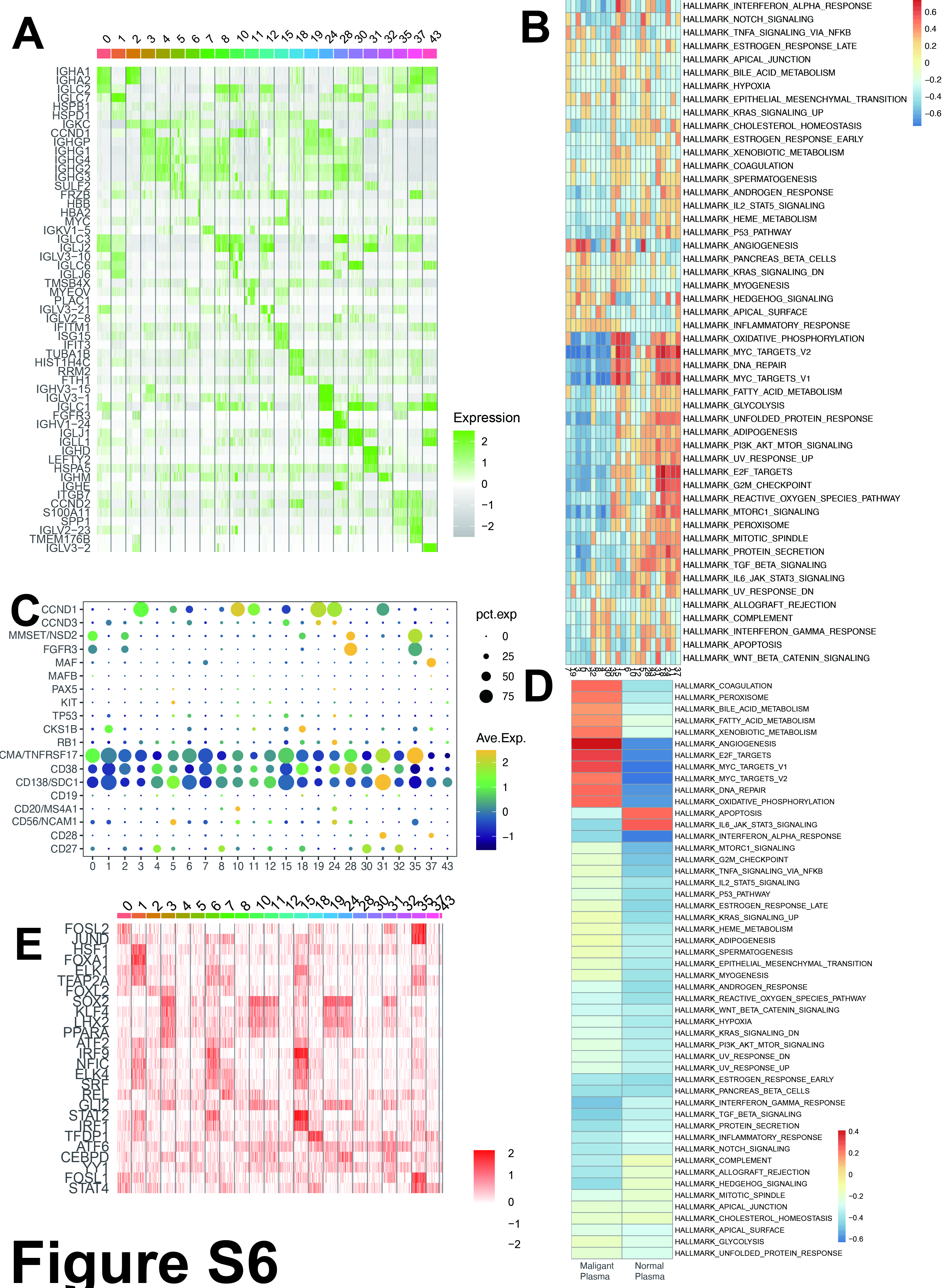

Supplement: Supplementary file 6 [file Image6.tif]

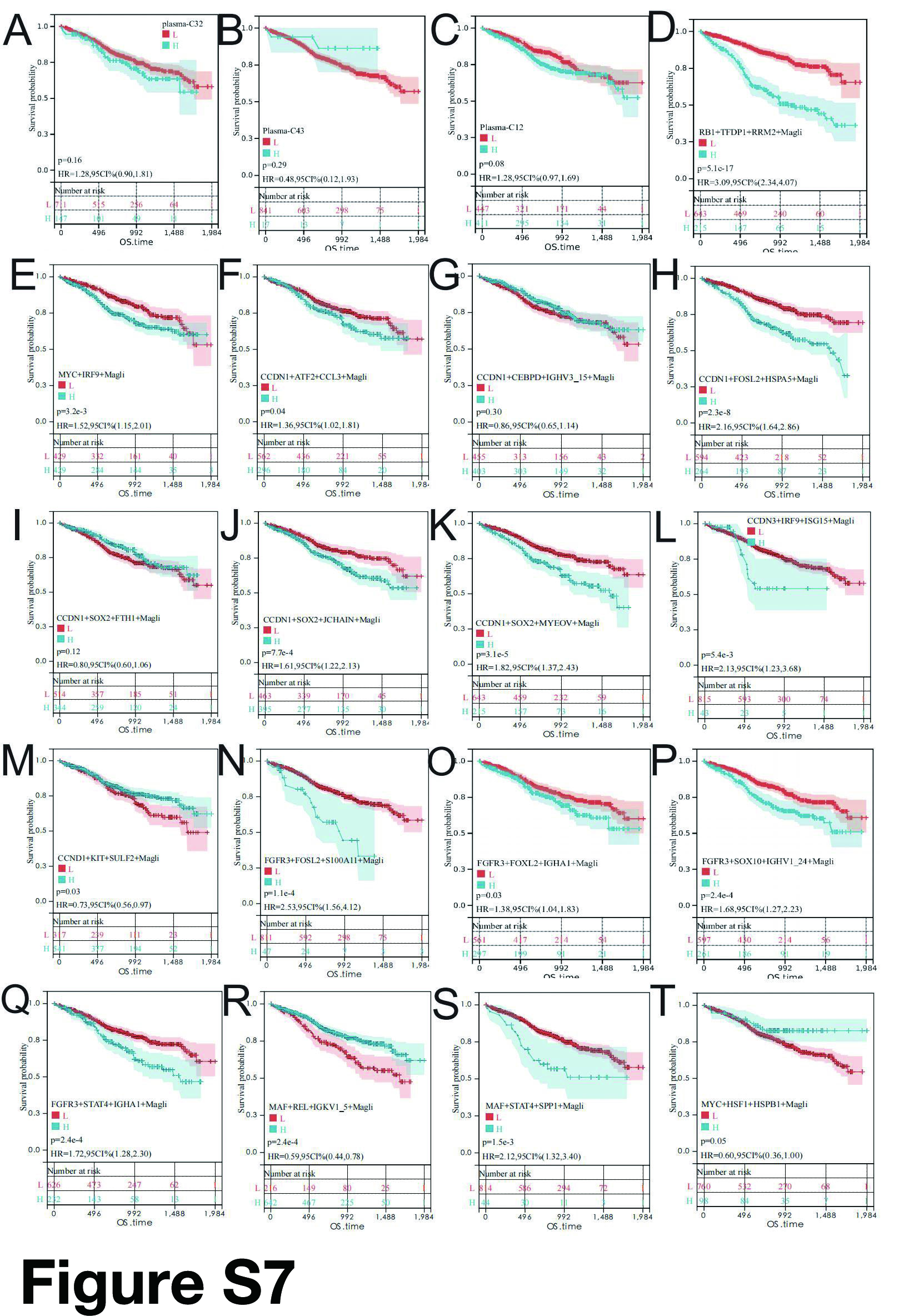

Supplement: Supplementary file 7 [file Image7.tif]

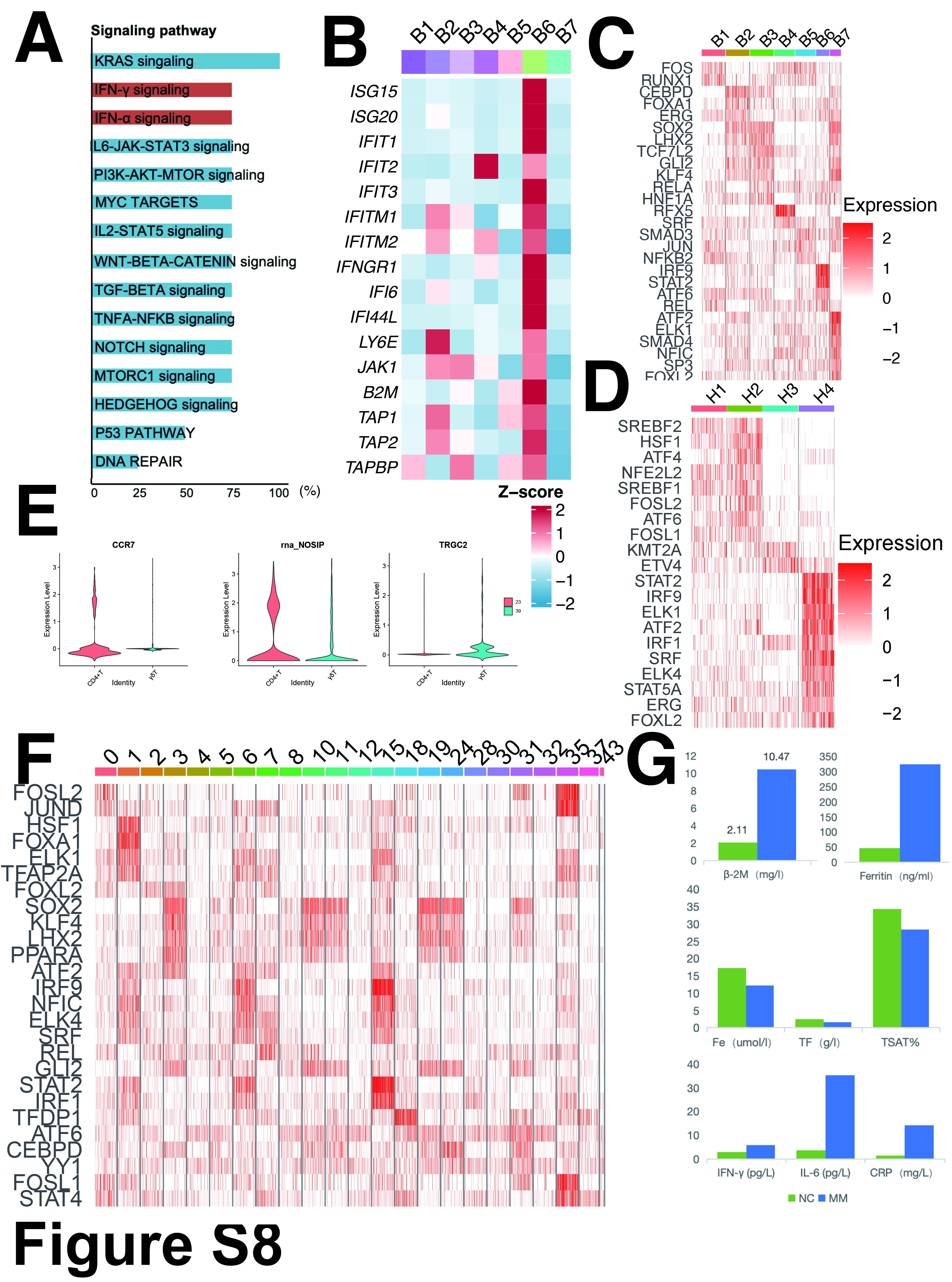

Supplement: Supplementary file 8 [file Image8.tif]

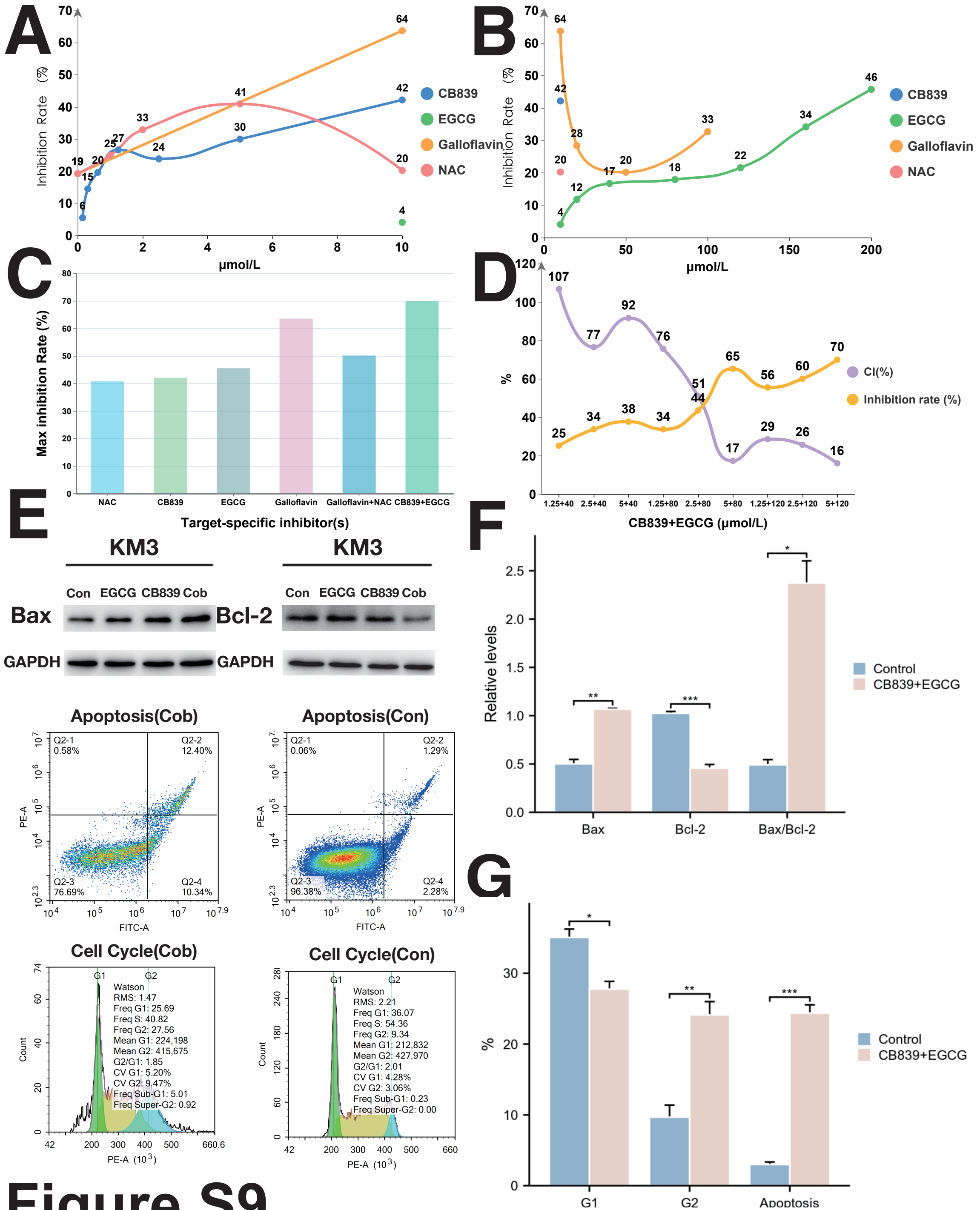

Figure S9

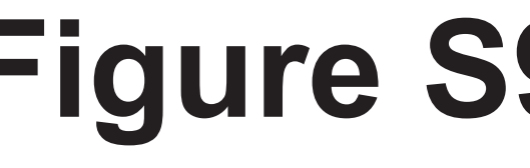

Supplement: Supplementary file 9 [file Image9.pdf]
