# Supplementary material for: Multi-omics reveals immune features in immune and non-immune cells, an IFN-γ/IFN-α-B2M positive feedback loop, and targeted metabolic therapy in multiple myeloma
Source: Front Immunol. 2025 Sep 8;16:1575079. doi: 10.3389/fimmu.2025.1575079 (PMC12450957; doi:10.3389/fimmu.2025.1575079)
Supplement: Supplementary file 11 [file Supplementaryfile1.docx]

## Material and methods

### 1.1 Single-cell RNA sequence analysis

### 1.1.1 Data Sources and Integration

Single-cell sequencing datasets, namely, GSE161195, GSE161722 and GSE117156, were downloaded from the Gene Expression Omnibus (GEO) database. The GSE161195 dataset contains the sequencing data of bone marrow-derived plasma cells (PCs) from 41 patients with primary refractory and early-relapsed MM, 11 healthy individuals and 15 patients with newly diagnosed MM. The sequencing platforms used were Illumina NextSeq 500 (*Homo sapiens*) and Illumina NovaSeq 6000 (*Homo sapiens*). The GSE161722 dataset contains the sequencing data of bone marrow aspirates from 6 patients with MM. The sequencing platform used was Illumina NovaSeq 6000 (*Homo sapiens*). The GSE117156 dataset contains the sequencing data of bone marrow-derived PCs from 29 patients with newly diagnosed PC neoplasms and 11 healthy individuals. Bone marrow-derived PCs were sorted via FACS at the single-cell level and were subjected to mRNA sequencing using the Illumina NextSeq 500 platform (*Homo sapiens*). Bulk RNA-seq dataset MMRF containing the survival information of patients with MM was downloaded from TCGA database. Eventually, the three GEO datasets were integrated through canonical correlation analysis using the Seurat package.

### 1.1.2 Cluster Analysis and Cell Annotation

The integrated data were uploaded to Seurat for QC analysis. Thereafter, principal component analysis (PCA) was performed, and the top 20 principal components and the top 3,000 variable genes were selected for subsequent analysis. The main cell clusters were identified using the FindClusters function (resolution = 0.8) in Seurat and visualised through t-distributed stochastic neighbour embedding (tSNE) analysis. The markers of each cell cluster were identified using the FindAllMarkers function, whereas the major cell types were recognised based on the markers obtained from the CellMarker database[1] and previous studies[2, 3].

### 1.1.3 Cell–cell Communication Analysis

Cell–cell communication at the molecular level was analysed using iTALK[4], which uses a cell–gene expression matrix from scRNA-seq as the input. The data matrix was processed according to the study design, and known ligands and receptors were selected, paired and functionally annotated using a built-in ligand–receptor database. The output was subsequently visualised in different formats using a data visualisation tool in iTALK.

### 1.1.4 Pseudo-time Trajectory Analysis

Monocle 2, an R package designed for analysing single-cell trajectories, was used to examine changes in immune cells during tumour progression[5]. The trajectories were visualised on 2D tSNE plots.

### 1.1.5 Functional Enrichment Analysis

Gene set variation analysis (GSVA) [6]was performed using 50 hallmark gene sets obtained from the Molecular Signatures Database as default sets. Gene set enrichment analysis (GSEA)[7] was performed to identify gene sets that were significantly enriched in MM cells. Both analyses were conducted using the GSVA and GSEA packages, respectively, and only gene sets with false discovery rate (FDR)-adjusted p-values of <0.05 and nominal p-values of <0.05 were considered significantly enriched.

### 1.1.6 Analysis of Transcription Factors

The DoRothEA package was used to examine the activity of transcription factors (TFs) by calculating the Viber score, thus obtaining TFs specific to each cell cluster.

**1.2 Single-cell metabolomics analysis**

Metabolic pathway activity was quantified using the scMetabolism R package, which computed scores for 67 pathways across single-cell clusters. Results were visualized via heatmap and nomogram. To identify pathways linked to plasma cell malignancy, Gene Set Enrichment Analysis (GSEA) and Gene Set Variation Analysis (GSVA) were performed. Additionally, key enzyme expression levels in glucose, lipid, and glutamine metabolism were assessed.

**1.3 Bulk RNA-seq analysis (Digital Flow Cytometry)**

CIBERSORTx[8] is a new machine-learning algorithm designed for detecting the abundance of cells in bulk RNA-seq data; this technique has been named ‘digital cytometry’. To evaluate the role of cells identified in this study, CIBERSORTx was used to analyse the gene expression profiles of patients in the MMRF cohort. Subsequently, KM analysis was performed based on the survival data of patients in the MMRF cohort.

**1.4 Multiplex Immunofluorescence Staining**

To perform multiplex immunofluorescence staining (mIF staining), paraffin-embedded tissue sections are first placed on slides and baked at 60°C for 30 minutes to ensure proper adhesion. The sections are then deparaffinized by immersing them in xylene for 10 minutes twice, followed by rehydration through a series of ethanol washes: 100% ethanol for 5 minutes twice, 95% ethanol for 5 minutes twice, 90%, 85%, 80%, and 75% ethanol, each for 5 minutes. After rehydration, the sections are rinsed twice with distilled water for 5 minutes each. Antigen retrieval is performed by microwaving the tissue in an antigen retrieval solution for 5 minutes at high power, followed by 15 minutes at low-medium power, allowing the slides to cool to room temperature. The slides are then washed three times with PBS for 5 minutes each. Non-specific binding is blocked by incubating the tissue with a blocking solution at room temperature for 10 minutes. After blocking, primary antibodies diluted to the working concentration are applied and incubated at 37°C for 1 hour, followed by three PBS washes for 5 minutes each. Secondary antibodies are then applied and incubated at 37°C for 10 minutes, followed by additional PBS washes. The fluorescent dye is added to the tissue for 5 minutes at room temperature, and the slides are washed three times with PBS. The antigen retrieval process is repeated to enhance signal strength. Nuclear staining is done using DAPI for 5 minutes at room temperature, followed by PBS washes. Finally, the slides are mounted with an anti-fade mounting medium to preserve fluorescence, and images are captured using a fluorescence or confocal microscope. Image analysis software is then used to perform quantitative analysis and statistical interpretation of the staining patterns, including the colocalization of different markers. Through mIF staining, we further validated the presence of these cell subpopulations.

**1.5 Machine Learning**

The logistic regression algorithm was used to incorporate all cell clusters (51 cell types) to construct model A.

Key cell clusters were identified using the lasso regression algorithm, in which lambda.min was used to construct model B. The scores of each cell cluster were multiplied by their corresponding coefficients and subsequently added to obtain the final risk score. Patients were divided into high- and low-risk groups based on the median risk score, and the risk scores of patients with different survival states were visualised.

**1.6 Drug Screening for Cellular Assays**

**1.6.1 Cell Counting Kit-8 Assay**

Malignant plasma cells exhibited profound metabolic alterations compared to their normal counterparts, including: (1) enhanced glycolytic flux, (2) upregulated OXPHOS activity, and (3) overexpression of glycolytic and glutaminolytic enzymes. The following metabolic inhibitors were selected: galloflavin (glycolysis/LDHA), CB-839 (glutaminolysis/GLS1), NAC (OXPHOS), and EGCG (glycolysis, glutaminolysis/GDH, OXPHOS). Two combination therapies were implemented: CB-839+EGCG and galloflavin+NAC. Their effects on the proliferation of the myeloma cell line KM3 were examined through in vitro assays.

The KM3 cell line was a generous gift from Professor Juan Du (Shanghai Changzheng Hospital, China). Cell proliferation was detected using the CCK8 kit (Meilunbio) according to the manufacturer’s instructions. NAC (HY-B0215) and galloflavin (HY-W040118) were purchased from MedChemexpress Ltd. (Shanghai, China). EGCG (S2250) and CB839 (S7655) were purchased from selleck (Shanghai, China)。

Cells were seeded in 96-well plates (2×10⁴cells/well) and treated with either single agents or combinations: EGCG (10-200μmol/L), CB-839 (0.15625-10μmol/L), galloflavin (0-100μmol/L), or NAC (0-10μmol/L), along with their respective combinations (CB-839+EGCG:1.25-5+40-120μmol/L; galloflavin+NAC: 20-100+2-10 μmol/L). After 48 h, cell viability was assessed by CCK-8 assay (10 μL/well, 37°C, 2-4 h) with 450 nm absorbance measurement, using 0.1% DMSO and drug-free medium as controls. Drug synergy was evaluated via Chou-Talalay combination index (CalcuSyn software), where CI<1, =1, and >1 indicated synergy, additivity, and antagonism respectively.

**1.6.2 Flow Cytometry**

Flow cytometry was implemented to detect the cell cycle distribution and apoptosis of KM3 cells. Cell cycle and apoptosis were assessed using Beyotime assay kits. Cells were collected into a centrifuge tube (15 mL) and fixed with 1 mL of precooled ethanol (70%) at 4 °C for 2 h. The cells were washed with phosphate buffered saline (PBS) and centrifuged with the supernatant removed. Subsequently, the cells were incubated with propyl iodide staining solution for 30 min at 37 °C in the dark, then the tubes were placed on ice and the cell distribution in G1, S, and G2 phases was evaluated using flow cytometry (Agilent) and analyzed using NovoExpress software. The Annexin V-FICT apoptosis detection kit (Beyotime) was used to evaluate apoptosis. Briefly, cells were collected into a centrifuge tube (15 mL), washed with PBS, and incubated with 5 µL Annexin V-FICT and 10 µL propyliodide for 20 min at room temperature in the dark. The tubes were placed on ice and apoptotic cells were measured using flow cytometer (Agilent) and analyzed using NovoExpress software.

**1.6.3 Western Blot Analysis**

Cells were lysed and the protein concentration was determined using the Bicinchoninic aci (BCA) protein quantification kit (Solarbio, Beijing, China). The proteins (30 µg protein per well) were separated by SDS-PAGE and transferred to PVDF membranes. The membranes were blocked in 5% bovine serum albumin (BSA) and incubated with primary antibodies (recombinant anti-Bax from Abcam and anti-Bcl-2 antibody from Santa) at 4 °C overnight, then washed and incubated with a secondary antibody at room temperature for 1 h. Subsequently, the protein bands were visualized using electro chemiluminescence (ECL) solution (Meilunbio).

**1.6.4 Statistical Analysis**

SPSS 25.0 software was used for the statistical analysis. The cell experimental data are presented as mean ± standard deviation (SD) and the student’s t-test was used to detect any differences between groups. *P* value < 0.05 showed significant difference and each cell experiment was repeated independently three times.

#### 2 Functional enrichment analysis and transcription factor analysis

#### 2.1 HSCs

H4 cells had upregulated interferon-alpha (IFN-α) and inflammatory responses and angiogenic effects. H3 cells had upregulated IFN-α and inflammatory responses. H1 and H2 cells (second differentiation pathway) had upregulated MYC , mTOR and NOTCH signalling and weak inflammatory responses, suggesting a high proliferative state and a suppressed inflammatory response.

**2.2 Myeloid cells**

#### 2.2.1 EPCs

In early EPCs (MEPCs), the MYC and angiogenic pathways were upregulated and metabolism was characterised by enhanced glycolysis. In intermediate EPCs, pathways associated with E2F signalling, MYC signalling, mitotic spindle, Hedgehog signalling, G2/M checkpoint, oxidative phosphorylation (OXPHOS), fatty acid metabolism and bile acid metabolism were upregulated. These pathways are related to cell proliferation, with the metabolic aspect characterised by enhanced lipid metabolism. In late EPCs, inflammatory responses and pathways associated with IL-2, IL-6, IFN-α, IFN-γ and other inflammatory factors were upregulated. TFs regulating E1 and E3 cells included GATA1 and RUNX2, whereas those regulating E2 cells included MYC, NR2C2 and USF1.

#### 2.2.2 Erythroblasts

In Ery1 cells, pathways associated with inflammatory responses, IL-2 signalling and TNFA signalling were upregulated. In Ery2 cells, cell proliferation pathways such as NOTCH, G2M and MYC pathways were significantly upregulated. In Ery3 cells, the angiogenic pathway was significantly upregulated. TFs regulating the Ery1 cluster included FOXL2 and USF1, whereas those regulating the Ery2 cluster included NR1H3, PPARA and IRF9. Additionally, the Ery3 cluster was regulated by ZEB1 (Figure S3C).

**2.2.3 Macrophages**

Pathways associated with angiogenesis, inflammation and cytokine signalling were upregulated in the M1 cluster. Pathways associated with hypoxia, P53 signalling, IL-6/JAK/STAT3 signalling, KRAS signalling, WNT signalling, NOTCH signalling, TGF signalling, apoptosis, interferon-gamma signalling and OXPHOS were upregulated in the M2 cluster. Pathways associated with MYC signalling, E2F signalling and fatty acid metabolism were upregulated in the Hy1 cluster. Pathways associated with IFN-α and peroxisomes were upregulated in the Hy2 cluster. The M1 cluster was regulated by SRF, PPARA and FOSL1, whereas the M2 cluster was regulated by RFX5 (Figure S3F).

**2.3 Lymphatic lineage**

**2.3.1 CD8+ T cells, CD4 T cells and γδ T cells**

In the Tex cluster, pathways associated with apoptosis, IL6/JAK/STAT3 signalling and cholesterol homeostasis were upregulated. In the Tn cluster, pathways associated with inflammatory responses and KRAS, NOTCH signalling were upregulated. In the Tem cluster, the angiogenic pathway was upregulated. In the Tc cluster, pathways associated with inflammatory responses, hypoxia, TNFA/NFKB signalling, IL2/STAT5 signalling, KRAS signalling, MYC signalling, NOTCH signalling and other immune response-related factors were upregulated. These pathways were found to play a major role in killing tumour cells.

Pathways associated with angiogenesis, TNFA/NFKB signalling, OXPHOS, NOTCH signalling, fatty acid metabolism, bile acid metabolism, KRAS signalling and hypoxia were upregulated in CD4 T cells (Figure S4D). TF analysis (Figure S4A) revealed that Tn cells were regulated by GATA1, FOXA1 and CEBPB; Tex cells were regulated by ASCL1, OTX2 and NFKB2; Tem cells were regulated by NFKB2 and Tc cells were regulated by GATA1. Pathways associated with WNT signalling, glycolysis, apoptosis and cholesterol metabolism were upregulated in γδ T cells. CD4 T cells were regulated by GATA1, FOXL2, SMAD4 and PPARA, whereas γδ T cells were primarily regulated by ATF6 (Figure S4E).

**2.3.2 B cells (excluding plasma cells)**

GSVA suggested that the IL6/JAK/STAT3, WNT and glycolytic pathways were upregulated in B1 cells; the MYC, IL6/JAK/STAT3 and glycolytic pathways were upregulated in B2 cells and the E2F, G2M and angiogenic pathways were upregulated in B3 cells. Pathways associated with E2F signalling, G2M checkpoint, OXPHOS, MYC signalling and TGF signalling were upregulated in B4 cells. The angiogenic pathway was upregulated in B5 cells. In addition, pathways associated with IFN-α signalling, IFN-γ signalling, hallmark protein secretion and unfolded protein response, P53 signalling, PI3K/AKT/MTOR signalling, EMT, angiogenesis and TNFA signalling were upregulated in B6 cells (Figure S5B).

TF analysis revealed that B1 cells were regulated by FOS and RUNX1, B2 cells were regulated by FOXA1 and CEBPD, B3 cells were regulated by SOX2 and LHX2, B4 cells were regulated by RFX5 and SRF, B5 cells were regulated by SMAD3 and JUN, B6 cells were regulated by IRF9 and STAT2 and B7 cells were regulated by ATF2 and ELK1 (Figure S5C)

**2.4 Stromal cells: fibroblasts and fibroblast progenitor cells**

According to the results of GSVA, pathways associated with angiogenesis, TGF signalling, hypoxia, inflammation, fatty acid metabolism, NOTCH signalling, OXPHOS, IL6/JAK/STAT3 signalling, PI3K/AKT/MTOR signalling and IFN-γ signalling were significantly upregulated in the F1 cluster. The MYC, E2F and MTORC1 pathways were upregulated in the F0 cluster. The Hedgehog and APICA pathways were upregulated in the F2 cluster. Pathways associated with angiogenesis, TNFA signalling, E2F signalling and IFN-α signalling were significantly upregulated in the F3 cluster (Figure S5E). F1 cells were mainly regulated by TFs such as NR2F2, ETV4, SP3 and SOX11, whereas F3 cells were mainly regulated by TFs such as FOXL2, GATA1, ATF2 and STAT4 (Figure S5F).

**References**

1. Zhang, X., et al., *CellMarker: a manually curated resource of cell markers in human and mouse.* Nucleic Acids Res, 2019. **47**(D1): p. D721-D728.

2. Mehtonen, J., et al., *Single cell characterization of B-lymphoid differentiation and leukemic cell states during chemotherapy in ETV6-RUNX1-positive pediatric leukemia identifies drug-targetable transcription factor activities.* Genome Med, 2020. **12**(1): p. 99.

3. Qin, P., et al., *Integrated decoding hematopoiesis and leukemogenesis using single-cell sequencing and its medical implication.* Cell Discov, 2021. **7**(1): p. 2.

4. Wang, Y., et al., *iTALK: an R Package to Characterize and Illustrate Intercellular Communication.* bioRxiv, 2019: p. 507871.

5. Qiu, X., et al., *Reversed graph embedding resolves complex single-cell trajectories.* Nat Methods, 2017. **14**(10): p. 979-982.

6. Hanzelmann, S., R. Castelo, and J. Guinney, *GSVA: gene set variation analysis for microarray and RNA-seq data.* BMC Bioinformatics, 2013. **14**: p. 7.

7. Subramanian, A., et al., *Gene set enrichment analysis: a knowledge-based approach for interpreting genome-wide expression profiles.* Proc Natl Acad Sci U S A, 2005. **102**(43): p. 15545-50.

8. Steen, C.B., et al., *Profiling Cell Type Abundance and Expression in Bulk Tissues with CIBERSORTx.* Methods Mol Biol, 2020. **2117**: p. 135-157.

Supplement Figure Legends

Figure S1 Metabolic Characteristics and Pathway Activities in MM

A: Metabolic Characteristics of Different Cell Types.

B: Comparison of Major Metabolic Pathway Activities in Individual Cells.

Figure S2

A: Violin maps demonstrating the expression of specific genes in HSC clusters

B: Pathways or gene sets commonly upregulated and downregulated in HSC clusters

C: Upregulated and downregulated TFs in HSC clusters

D：Violin maps demonstrating the expression of specific genes in EPC clusters

E：Pathways or gene sets commonly upregulated and downregulated inEPC clusters

F：Upregulated and downregulated TFs in EPC clusters

Figure S3

A: Violin maps demonstrating the expression of specific genes in erythroblast clusters

B: Heat map demonstrating the expression of myeloid-related genes in erythroblast clusters

C: Upregulated and downregulated TFs in erythroblast clusters

D: Violin maps demonstrating the expression of specific genes in Macrophage clusters

E: Heat map demonstrating the expression of immune checkpoints in Macrophage clusters

F: Upregulated and downregulated TFs in Macrophage clusters

Figure S4

A: Upregulated and downregulated TFs in CD8+T cell clusters

B: Pathways or gene sets commonly upregulated and downregulated in CD8+T cell clusters

C: Violin maps demonstrating the expression of specific genes in CD4+T and γδT

D: Pathways or gene sets commonly upregulated and downregulated in CD4+T and γδT

E: Upregulated and downregulated TFs in CD4+T and γδT

Figure S5

A: Violin maps demonstrating the expression of specific genes in B cell clusters

B: Pathway or gene sets commonly upregulated and downregulated among clusters of B cell

C: Upregulated and downregulated TFs in B cell clusters

D: Violin maps demonstrating the expression of specific genes in fibroblast clusters

E: Pathway or gene sets commonly upregulated and downregulated among clusters of fibroblast

F: Upregulated and downregulated TFs among clusters of fibroblast

Figure S6

A:Expression heat map of top five genes in MM cell clusters

B:Pathways or gene sets commonly upregulated and downregulated in MM cell clusters

C: Mutation expression profile and immunophenotype in MM cell clusters

D: Pathway or gene sets commonly upregulated and downregulated among clusters of normal PCs and malignant PCs

E: Upregulated and downregulated TFs among clusters of MM cell

Figure S7 The correlation between the relative abundance of different Subsets of plasma cells and overall survival was examined using the CIBERSORTx algorithm

Figure S8: Signaling Pathways and MM Cell Interactions

A: Signaling pathway upregulation frequency across all cell types in cell-cell interaction network
B: ISG15+ B cells express IFNGR1
C: ISG15+ B cells express IRF9 and STAT2
D: H4 cells express IRF1, IRF9, and STAT2
E: γδ T cells express TRGC2
F: ISG15+ MMCs express IRF1, IRF9, and STAT2
G: Validation cohort analysis of serum levels of β-2M, ferritin, serum Fe, transferrin, TSAT, IFN-γ, IL-6, and CRP in MM patients vs. controls

.

**Figure S9: Inhibitory Effects of CB839, EGCG, Galloflavin, NAC on** **MM** **Cells**

A&B: Inhibitory effects of **CB839**, **EGCG**, galloflavin, and **NAC** on **MM** cells
C: Max inhibitory effects of **CB839**, **EGCG**, galloflavin, **NAC**, galloflavin+**NAC**, **CB839**+**EGCG** on **MM** cell proliferation
D: **Combination index** plot (CI<1=synergy, CI=1=additive, CI>1=antagonistic)
E: **Flow cytometry** and **Western blot** analysis of **BCL-2/Bax** protein expression, apoptosis, and cell cycle changes in combination vs control groups. Control(Con),**CB839**+**EGCG (Conbined,Con)**
F: Differences in Bax, Bcl-2 expression and Bax/Bcl-2 ratio between combination and control groups. * indicates P<0.05, ** indicates P<0.01, *** indicates P<0.001.

G: Differences in **G1**, **G2**, and apoptosis between combination and control groups.* indicates P<0.05, ** indicates P<0.01, *** indicates P<0.001.

Figure S10: Tumor Microenvironment of **MM**

A: Overview of ligand-receptor interactions between cell clusters
B: Interaction map of cell clusters in **MM** **TME**
C: **mIF** staining confirming **MEPCs** presence in **TME**
D: **mIF** staining confirming **MDSC** presence in **TME**
E: **mIF** staining confirming **M2** macrophage presence in **TME**
F: **mIF** staining confirming **Tex** and γδ T cell presence in **TME**
G: **mIF** staining confirming **ISG15+** B cell presence in **TME**
H: **mIF** staining confirming **ISG15+** B cell presence in **TME**
I: **mIF** staining confirming **CAFs** presence in **TME**
J: Quantification of immune cell populations showing changes between relapse (**RE**) and initial diagnosis (**ID**) with shifts in **MEPCs**, **MDSC**, **M2**, **Tex**, γδ T cells, **ISG15+** B cells, and **CAFs**
