# Supplementary material for: Multi-omics reveals immune features in immune and non-immune cells, an IFN-γ/IFN-α-B2M positive feedback loop, and targeted metabolic therapy in multiple myeloma
Source: Front Immunol. 2025 Sep 8;16:1575079. doi: 10.3389/fimmu.2025.1575079 (PMC12450957; doi:10.3389/fimmu.2025.1575079)
Supplement: Supplementary file 12 [file Table1.docx]

GSE161195 https://www.ncbi.nlm.nih.gov/geo/query/acc.cgi?acc=GSE161195

GSE161722 https://www.ncbi.nlm.nih.gov/geo/query/acc.cgi?acc=GSE161722

GSE117156 <https://www.ncbi.nlm.nih.gov/geo/query/acc.cgi?acc=GSE117156>

TCGA MMRF cohort <https://gdc.cancer.gov/>

https://gdc.cancer.gov/about-gdc/contributed-genomic-data-cancer-research/foundation-medicine/multiple-myeloma-research-foundation-mmrf
