# Supplementary material for: Multi-omics reveals immune features in immune and non-immune cells, an IFN-γ/IFN-α-B2M positive feedback loop, and targeted metabolic therapy in multiple myeloma
Source: Front Immunol. 2025 Sep 8;16:1575079. doi: 10.3389/fimmu.2025.1575079 (PMC12450957; doi:10.3389/fimmu.2025.1575079)
Supplement: Supplementary file 10 [file Image10.pdf]

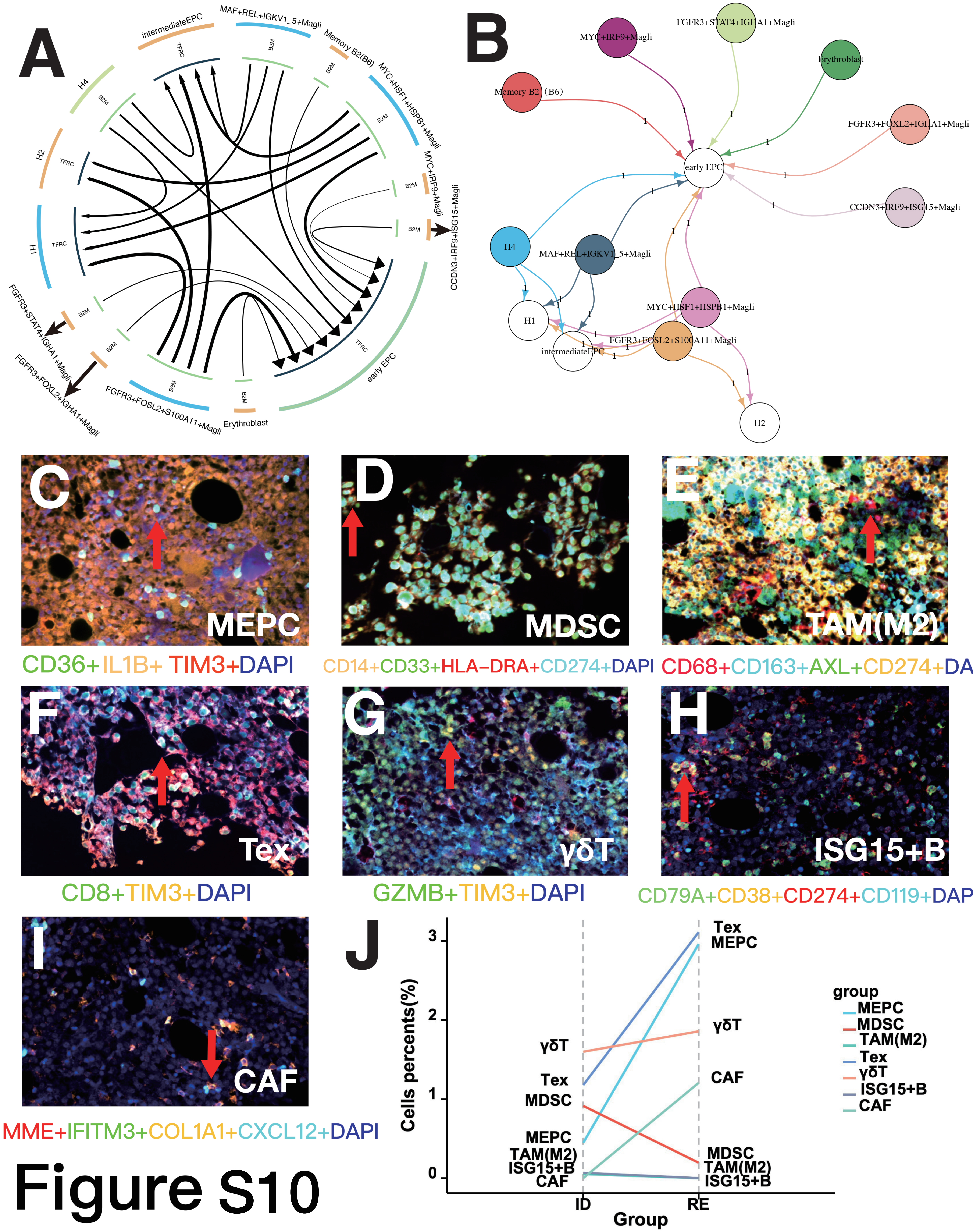
A
intermediateEPC
MAF+REL+IGKV1_5+Magli
Memory B2(B6)
MYC+HSF1+HSPB1+Magli
MYC+IRF9+Magli
CCDN3+IRF9+ISG15+Magli
early EPC
Erythroblast
FGFR3+FOSL2+S100A11+Magli
FGFR3+FOXL2+IGHA1+Magli
FGFR3+STAT4+IGHA1+Magli
H1
H2
H4
TFRC
B2M
B
MYC+IRF9+Magli
FGFR3+STAT4+IGHA1+Magli
Erythroblast
Memory B2 (B6)
FGFR3+FOXL2+IGHA1+Magli
early EPC
CCDN3+IRF9+ISG15+Magli
H4
MAF+REL+IGKV1_5+Magli
H1
MYC+HSF1+HSPB1+Magli
intermediateEPC
FGFR3+FOSL2+S100A11+Magli
H2
C
MEPC
CD36+IL1B+ TIM3+DAPI
D
MDSC
CD14+CD33+HLA−DRA+CD274+DAPI
E
TAM(M2)
CD68+CD163+AXL+CD274+DA
F
Tex
CD8+TIM3+DAPI
G
γδT
GZMB+TIM3+DAPI
H
ISG15+B
CD79A+CD38+CD274+CD119+DAP
I
CAF
MME+IFITM3+COL1A1+CXCL12+DAPI
J
Cells percents(%)
Group
ID
RE
γδT
Tex
MDSC
MEPC
TAM(M2)
ISG15+B
CAF
group
MEPC
MDSC
TAM(M2)
Tex
γδT
ISG15+B
CAF

Figure S10
